# Synthesis of New Polyheterocyclic Pyrrolo[3,4-*b*]pyridin-5-ones via an Ugi-Zhu/Cascade/Click Strategy

**DOI:** 10.3390/molecules28104087

**Published:** 2023-05-14

**Authors:** Roberto E. Blanco-Carapia, Enrique A. Aguilar-Rangel, Mónica A. Rincón-Guevara, Alejandro Islas-Jácome, Eduardo González-Zamora

**Affiliations:** 1 Departamento de Química, Universidad Autónoma Metropolitana-Iztapalapa, Av. Ferrocarril San Rafael Atlixco 186, Col. Leyes de Reforma 1A Sección, Iztapalapa, Mexico City 09310, Mexico; edreyblanco@gmail.com (R.E.B.-C.); enrique.alejandro.aguilar.19@gmail.com (E.A.A.-R.); 2 Departamento de Biotecnología, Universidad Autónoma Metropolitana-Iztapalapa, Av. Ferrocarril San Rafael Atlixco 186, Col. Leyes de Reforma 1A Sección, Iztapalapa, Mexico City 09310, Mexico; monicarinconguevara@gmail.com

**Keywords:** multicomponent reactions (MCRs), Ugi-Zhu reaction, click chemistry, polyheterocycles, pyrrolo[3,4-*b*]pyridin-5-ones, 5-substituted-1*H*-tetrazoles, 2,4-diamino-1,3,5-triazines

## Abstract

A diversity-oriented synthesis (DOS) of two new polyheterocyclic compounds was performed via an Ugi-Zhu/cascade (*N*-acylation/*aza* Diels-Alder cycloaddition/decarboxylation/dehydration)/click strategy, both step-by-step to optimize all involved experimental stages, and in one pot manner to evaluate the scope and sustainability of this polyheterocyclic-focused synthetic strategy. In both ways, the yields were excellent, considering the high number of bonds formed with release of only one carbon dioxide and two molecules of water. The Ugi-Zhu reaction was carried out using the 4-formylbenzonitrile as orthogonal reagent, where the formyl group was first transformed into the pyrrolo[3,4-*b*]pyridin-5-one core, and then the remaining nitrile group was further converted into two different nitrogen-containing polyheterocycles, both via click-type cycloadditions. The first one used sodium azide to obtain the corresponding 5-substituted-1*H*-tetrazolyl-pyrrolo[3,4-*b*]pyridin-5-one, and the second one with dicyandiamide to synthesize the 2,4-diamino-1,3,5-triazine-pyrrolo[3,4-*b*]pyridin-5-one. Both synthesized compounds may be used for further in vitro and in silico studies because they contain more than two heterocyclic moieties of high interest in medicinal chemistry, as well as in optics due to their high π-conjugation.

## 1. Introduction

**Figure 1:**
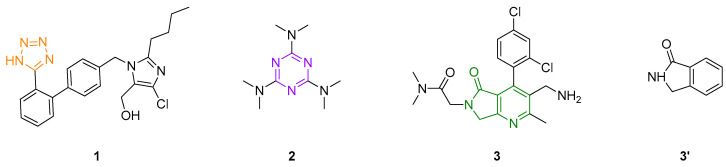
Losartan (**1**), altretamine (**2**), BMS-76778 (**3**), and isoindolin-1-one (**3′**).

Nitrogen-containing heterocycles play a very important role in medicinal chemistry because they are the structural core of a broad spectrum of biologically active molecules. Some are in preclinical trials, and many others are already commercially available [1]. Among the most privileged *N*-heterocycles are the 5-substituted-1*H*-tetrazoles (5S-1*H*-Ts), due mainly to their ability to surrogate the carboxylic acids (RCOOH) into living systems since they have close values of pKa (5S-1*H*-Ts: 4.5 to 4.9, RCOOH: 4.2 to 4.4), similar topologies, and practically the same electrostatic potential and electron density [2]. For these reasons, 5S-1*H*-Ts are considered bioisosteres of carboxylic acids [3]. In addition, 5S-1*H*-Ts are at least ten times more lipophilic compared to carboxylic acids, a property that makes them even more valuable for designing new drug candidates with enhanced pharmacokinetic profiles [4]. Indeed, to date 23 drugs containing the 5S-1*H*-T system have been approved by the FDA for safe use in humans, including antivirals, antiallergics, analgesics, anti-inflammatories, and especially antihypertensives such as the blockbuster drug losartan (**1**, Figure 1) [5]. Another *N*-heterocycle of high interest in medicinal chemistry, due also to its presence in many bioactive compounds and some marketed drugs such as the anticancer alkylating agent altretamine (**2**, Figure 1) [6], is the 1,3,5-triazine, known as *s*-triazine [7]. This compound is a six-membered aromatic heterocycle containing three nitrogen atoms that are able to form strong H-bonds, and three C-*sp^2^* positions that can be decorated a la carte [8]. Finally, the pyrrolo[3,4-*b*]pyridin-5-one is a fused-type *bis*-heterocycle that is also of high interest in medicinal chemistry. It includes the antidiabetic agent BMS-767778 (**3**, Figure 1) [9] which is an N-*sp^2^*-containing surrogate of the naturally occurring isoindolin-1-one (**3′**, Figure 1) [10]. The pyrrolo[3,4-*b*]pyridin-5-one system is very special in our research group. It has been the most essential molecule because it can be smartly assembled in a one-pot manner using multicomponent reactions (MCRs).

MCRs are privileged synthetic tools that involve sequential additions of at least three reagents into the same reactor (one pot). Given the nature of MCRs, they can be accessed quickly, in comparison to linear synthesis, to a series of highly functionalized and structurally complex products, leading to high atomic economy, eventually in good chemical yields and in short reaction times, needing only one work-up and in most cases just one purification process [11]. MCRs (together with click reactions) are considered one of the most robust synthetic methodologies because a large variety of polyheterocyclic compounds of high interest in medicinal chemistry and optics have been synthesized in record time, satisfying the majority of the 12 principles of green chemistry [12].

**Scheme 1:**
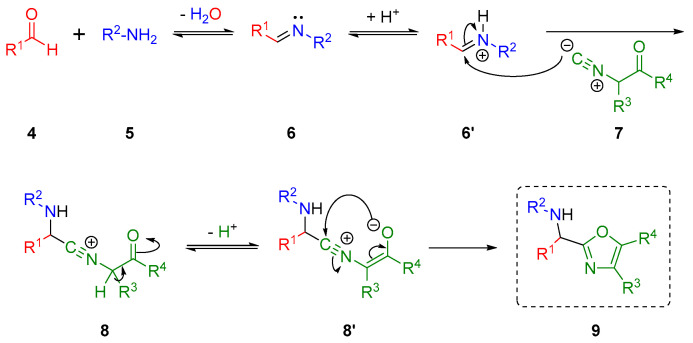
Mechanism of the UZ-3CR.

In 2001, J. Zhu and co-workers reported a new and elegant variant of the truncated Ugi reaction, recently recognized by us as the Ugi-Zhu three-component reaction (UZ-3CR) [13], where primary amines react sequentially with aldehydes, and isocyanoacetamides (derived from amino acids) to lead to trisubstituted 5-aminooxazoles under mild reaction conditions [14]. The most plausible reaction mechanism consists of the condensation between aldehydes **4** with amines **5** to give rise to the corresponding Schiff bases **6**, which, depending on the substituents (**R_1_** and **R_2_**), may or may not require activation by an acid, thus favoring the α-nucleophilic attack of the isocyanides **7**, leading to formation of nitrilium cations **8**. Then, the intermediate **8′** rapidly tautomerizes via a non-prototropic chain-ring process to the corresponding oxazoles **9** (Scheme 1).

Thus, as a part of our ongoing program to synthesize novel and complex polyheterocyclic compounds, we have developed several methodologies based on UZ-3CR as key synthetic tool for preparing series of pyrrolo[3,4-*b*]pyridin-5-ones either bound, fused or linked to various heterocyclic frameworks of interest in optics and medicinal chemistry. However, for the present work, the synthetic strategy behind a couple of new *tris*-heterocycles, from the diversity-oriented synthesis (DOS) approach, started with an Ugi-Zhu reaction performed stepwise to first obtain a Schiff base, and then, the corresponding 5-aminooxazole. This pivotal heterocycle then reacts with maleic anhydride via a cascade process (*aza* Diels–Alder cycloaddition/*N*-acylation/decarboxylation/dehydration) to assemble the pyrrolo[3,4-*b*]pyridin-5-one core [15]. Finally, click reactions with different reagents and conditions provide the polyheterocyclic products.

## 2. Results and Discussion

**Scheme 2:**
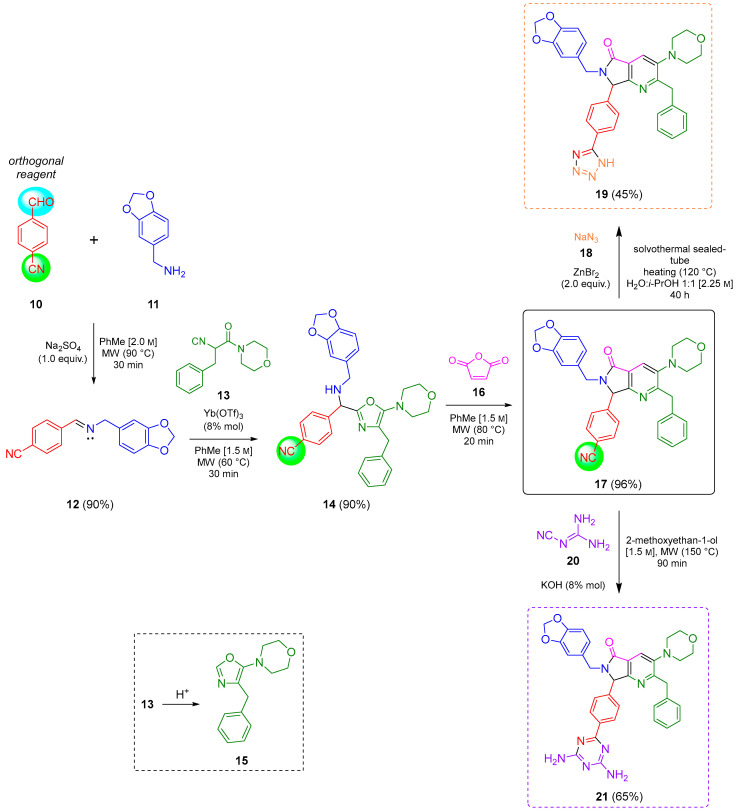
General scheme of synthesis.

The reaction sequence started with the synthesis of the Schiff base **12** via a condensation of the 4-formylbenzonitrile (**10**) with piperonylamine (**11**). It is worthy to note that the reagent **10** contains two functional groups, aldehyde, and nitrile. The first one is used in the UZ-3CR, and the second in further click reactions. Indeed, **10** is considered an orthogonal reagent. Thus, some experiments were performed with the aim to find the optimal reaction conditions. The first entry was carried out at room temperature, under continuous stirring, without additives or catalysts, and using methanol as solvent. The reaction was monitored with thin layer chromatography (TLC) until the amount of aldehyde in the reaction mixture was consumed or considerably decreased. However, after 24 hours, there was no appreciable change. Therefore, some additional experiments were performed with varying temperatures, additives, and solvents. The optimal conditions turned out to be using anhydrous toluene as solvent at 2.0 M concentration, one equivalent of anhydrous sodium sulfate as drying agent, and under microwave heating conditions at 90 °C for 30 min (see Table S1 in the Supplementary Material for further details). It is essential to highlight that the addition of anhydrous sodium sulfate to the reaction mixture in the formation of the Schiff base allowed obtaining the condensation product **12** in 90% yield. This dehydrating salt is especially useful when reactions assisted by microwaves are carried out in closed-vessel systems, where Dean–Stark traps cannot be implemented. The next step was a nucleophilic α-addition of the isocyanide **13** [16] to the imine **12** to provide the key 5-aminoxazole **14**. The first tests behind optimal reaction parameters were carried out under relatively mild conditions since it was found previously that isocyanide **13** can undergo an acid-catalyzed process of chain-ring tautomerization leading to the 5-aminooxazole **15** as by-product. However, the desired 5-aminooxazole **14** was not detected by TLC. Thus, by increasing the temperature, traces of **14** were observed but the main isolated compound was **15**, again. In this context, Lewis acids were used to activate the imine **12**, so catalytic amounts of a panel of them in different proportions were added, and the reaction mixture was heated at 60 °C for 5 min before addition of the isocyanide **13**. Then, the new resulting mixture was heated for 25 minutes using microwaves as heat source. The use of ytterbium triflate allowed obtaining the 5-aminooxazole **14** with the best yield (90%). The substantial improvement in the performance using ytterbium (III) compared to the other Lewis acids is because it is the most polarizable due to its size compared to scandium (III), indium (III), and aluminum (III). Indeed, it is a softer acid according to the HSAB Pearson principle, and therefore, it coordinates preferentially with the imine, activating it better. It is worth mentioning that experimentally, it was found that both indium chloride and scandium triflate reach coordination with isocyanide and even with the nitrile group, avoiding an efficient coordination with the imine (see Table S2 in the Supplementary Material for further details). Then, the 5-aminoxazole **14** was reacted with maleic anhydride (**16**) into a cascade process (*aza* Diels-Alder cycloaddition/*N*-acylation/decarboxylation/dehydration) to generate the corresponding pyrrolo[3,4-*b*]pyridin-5-one **17**. Different entries were carried out using toluene as solvent but varying temperature, finding that at 80 °C in 20 minutes the reaction was accomplished in 96% yield (see Table S3 in the Supplementary Material for further details). It is worth mentioning that a couple of additional experiments were performed at temperatures just over 80 °C but decomposition and formation of many uncharacterized by-products were observed, in addition to a very difficult work-up. The next step was the transformation of nitrile group from the pyrrolo[3,4-*b*]pyridin-5-one **17** into two nitrogenated heterocycles, tetrazole and triazine, both via click reactions. Thus, it has been widely documented that the most common reactions to synthesize 5-substituted-1*H*-tetrazoles are the (3+2) cycloadditions [17] between nitriles and some azide source as the 4π-component, catalyzed by Bronsted or Lewis acids [18,19,20,21]. In addition, it is known that aromatic nitriles as 2π-components in [3+2] cycloadditions react very low, unless they have strong EWGs [22], or if the [3+2] cycloadditions are catalyzed by acids. One of the rare examples of non-catalyzed [3+2] cycloadditions involving 4π-components to nitriles (2π-components) was reported by Jasinski and co-workers in 2020 [23]. In the compound **17**, the nitrile group is placed in the *para*-position relative to a methine group, so the [3+2] cycloaddition occurred due to a less steric hindrance, but it was not easy. Thus, a first attempt was carried out using the methodology described by Harasawa and co-workers in 2013 [24], which is useful for the synthesis of 5-substituted-1*H*-tetrazoles from electronically deactivated aromatic nitriles with some azide source. Unfortunately, the reaction did not proceed, probably due to a protonation of the N(*sp^2^*)-atom from pyridine **17**. Therefore, the strategy was changed, and the methodology published in 2001 by Demko and Sharpless was used, employing ZnBr_2_ in equimolar amounts, and sodium azide (**18**) [25] in a mixture 1:1 *v*/*v* of H_2_O/*i*-PrOH at reflux using conventional heating, or under microwave heating conditions. To our delight, the desired polyheterocycle **19** was obtained in both cases but in low yields, 9% and 6%, respectively, in addition to the fact that the work-up became laborious. Based on these results, it was considered that more drastic conditions were required to improve the yield, so the reaction was repeated, but using a pressure sealed tube. These conditions allowed performance of the cycloaddition but using a solvothermal sealed tube. Thus, the corresponding compound **19** was obtained in 45% yield, and, with respect to the work-up, it became trivial because once the reaction was finished, the tube reactor was left to cool, and a mixture of hexanes/EtOAc led to the precipitation of tetrazole. Finally, the polyheterocycle **19** was washed with ethyl acetate and hot water and dried under high vacuum (see Table S4 in the Supplementary Material for further details). Finally, the preparation of a second polyheterocycle also from the chemical scaffold **17** consisted of the annulation reaction between **17** and dicyandiamide (**20**) via a [3+2] cycloaddition assisted by microwave radiation. Thus, the reaction was carried out using DMSO as solvent and KOH as catalyst, according to the methodology reported by Fang and co-workers [26]. For the first attempt at 100 °C, the reaction did not proceed, so, the temperature was increased to 125 °C, and 150 °C but, in both cases only traces of the product **21** were detected. It is worth mentioning that from 150 °C, the DMSO decomposes into methane, sulfur dioxide, and other volatile substances [27,28]. The reaction was repeated, replacing the DMSO by 2-methoxyethan-1-ol at 150 °C again, but this time the desired product **21** was obtained in 65% yield, Scheme 2 (see Table S5 in the Supplementary Material for further details).

**Scheme 3:**
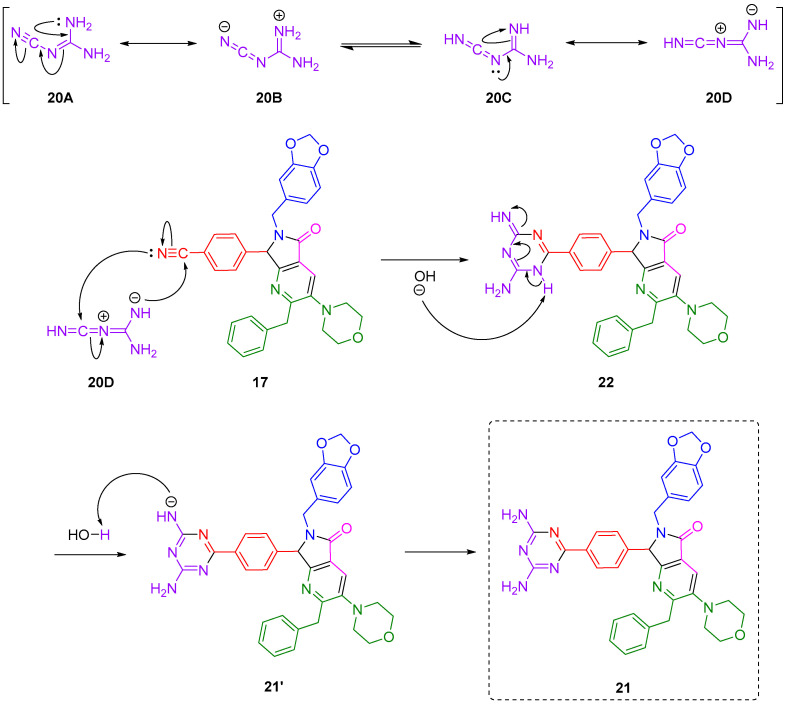
Plausible reaction mechanism for the click reaction between dicyandiamide and nitrile group from pyrrolo[3,4-*b*]pyridine-5-one via a click-type (4+2) cycloaddition.

Although there are some reports about the synthesis of triazines from nitriles and dicyandiamide via [4+2] cycloadditions, to our best knowledge, there is no proposals of mechanisms. Thus, in Scheme 3, a plausible reaction mechanism is proposed. Dicyandiamide (**20**) exists in different canonic forms, with its zwitterionic form **20D** the one that carries out the intermolecular [4+2] cycloaddition, leading to the formation of the six-membered polyheterocycle **22**. This intermediate subsequently undergoes a base-assisted aromatization to get finally the 2,4-diamino-1,3,5-triazine-pyrrolo[3,4-*b*]pyridin-5-one **21**, after recovering a H-atom from water. It is noteworthy that despite Scheme 3 suggesting a synchronous one step mechanism between **20D** and **17**, in fact the [4+2] cycloadditions can occur stepwise, even by non-polar [29], or polar [30] mechanisms. As a matter of fact, all kind of Diels–Alder cycloadditions, and, in general click reactions, remain controversial mainly due to their various possible reaction mechanisms. In this way, theoretical and experimental studies behind reaction mechanisms will be worthy of research.

**Scheme 4:**
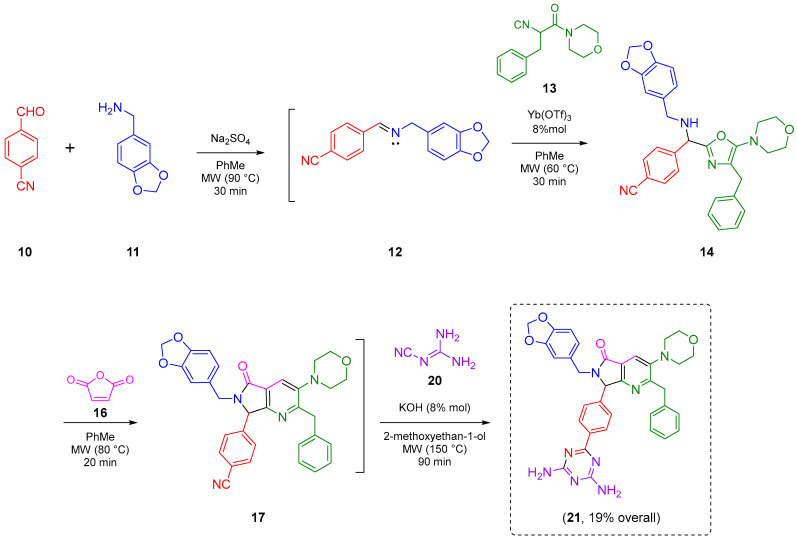
One pot synthesis of 2,4-diamino-1,3,5-triazine-pyrrolo[3,4-*b*]pyridin-5-one **21** via a domino process Ugi-Zhu/*aza* Diels–Alder/*N*-acylation/decarboxylation/dehydration/[4+2] cycloaddition.

Having the optimal conditions for the step-by-step synthesis of compounds **12**, **14**, **17** and **21**, and with the idea of evaluating the robustness of this methodology from the one pot chemistry approach, the synthesis of the final compound **21** was carried out via a domino process coupling in sequential manner the Ugi-Zhu reaction with the *N*-acylation, decarboxylation, dehydration, and the [4+2] cycloaddition, yielding 19%. Of course, this yield seems to be low, but it is not true at all considering the high number of formed bonds, the atom economy (89%), and that only a couple of water molecules and one carbon dioxide were released in the entire domino process (Scheme 4). In addition, only one work up and purification process was needed. In the same way, only one solvent shift was performed, but by evaporation/addition, the reaction mixture was maintained in the same MW-sealed reaction tube.

## 3. Materials and Methods

### 3.1. General Information, Instrumentation, Software and Chemicals

^1^H and ^13^C nuclear magnetic resonance (NMR) spectra were acquired on a Bruker AMX Advance III spectrometer (500 MHz, Fällande, Uster, Switzerland). The solvents used for NMR experiments were deuterated chloroform (CDCl_3_) and deuterated dimethyl sulfoxide (DMSO-*d_6_*). Chemical shifts are reported in parts per million (d/ppm). Coupling constants are reported in Hertz (J/Hz). The internal reference for NMR spectra was tetramethyl silane (TMS) at 0.00 ppm. Multiplicities of the signals are reported using the standard abbreviations: singlet (s), doublet (d), triplet (t), quartet (q), and multiplet (m). NMR spectra were analyzed using the MestReNova software Ver. 12.0.0-20080 (A Coruña, Spain). The infrared (IR) spectrum was acquired on a Perkin-Elmer 2000 spectrometer (Norwalk, CT, USA) using the attenuated total reflectance (ATR) method. The maximum absorbance peaks are reported in reciprocal centimeters (ν_max_/cm^−1^), uncorrected. The IR spectrum was analyzed using the Origin software (Ver. 2018b, 9.55, OriginLab Corporations, Northampton, MA, USA). The high-resolution mass spectroscopy (HRMS) spectrum was acquired by electrospray ionization (ESI) on a Micro-TOF II spectrometer Bruker Daltonics GmbH (Bremen, Germany). The sample was injected directly (Apollo source) and analyzed by the time-of-flight method (TOF). The HRMS spectrum was analyzed using the Compass software (Ver. 1.5, Bruker Daltonik GmbH, Bremen, Germany). Microwave-assisted reactions were performed in closed-vessel mode on a CEM Discover SP MW-reactor (Matthews, North Carolina, CA, USA). Reaction progress was monitored by thin-layer chromatography (TLC), and the spots were visualized under ultraviolet (UV) light (254 or 365 nm). Glass preparative plates (20 × 20 cm) coated with silica-gel 60 doped with UV indicator (F_254_) were used to purify the products. All starting reagents and solvents were used as received (without further purification, distillation, or dehydration). Chemical structures were drawn using the ChemDraw software (Ver. 15.0.0.106 Professional, Perkin Elmer Informatics, Cambridge, MA, USA). The purity of all synthesized products (>96%) was assessed by NMR.

### 3.2. Synthesis of 4-(((Benzo[d] [1,3]dioxol-5-ylmethyl)imino)methyl)imino)methyl)benzonitrile ***12***

The 4-formylbenzonitrile (1.0 mmol, 1.0 equiv.), 1,3-benzodioxole-5-methylamine (1.0 mmol, 1.0 equiv.) and sodium sulfate anhydrous (1.0 mmol, 1.0 equiv.) were placed in a sealed CEM Discover microware reaction tube (10 mL) and diluted in anhydrous toluene (1.5 mL). The mixture was stirred and heated using microwave irradiation (90 °C, 150 W) for 30 min. Subsequently, the reaction mixture was filtered, and the resulting solid was washed with 15 mL of dichloromethane and 15 mL of acetone; then, it was dried under high vacuum, obtaining (237.86 mg, 90% yield) of a light yellow solid, R*_f_* = 0.04 (EtOAc); ^1^H NMR (500 MHz, CDCl_3_): δ 8.37 (s, 1H, H-12), 7.86 (d, 2H, H-17, H-15, *J* = 8.0 Hz), 7.69 (d, 2H, H-18, H-14, *J* = 8.0 Hz), 6.84–6.76 (m, 3H, H-6, H-7, H-9), 5.94 (s, 2H, H-2), 4.76 (s, 2H, H-10) ppm; ^13^C NMR (125 MHz, CDCl_3_): δ 159.6 (C-12), 147.8 (C-4), 146.8 (C-5), 139.9 (C-13), 132.4 (C-15, C-17), 132.3 (C-8), 128.6 (C-14, C-18), 121.4 (C-7), 118.4 (C-19), 114.0 (C-16), 108.6 (C-6), 108.3 (C-9), 101.0 (C-2), 64.8 (C-10) ppm; HRMS: (ESI^+^) *m/z* calcd. for [M − H]^+^ C_16_H_13_N_2_O_2_^+^ 265.0972, found 265.0968 (error = 1.3 ppm); FT-IR ($\nu ,cm^{-1}$): 2835 (aryl H), 2242 (C≡N), 1480, 1442 (C=N), 1362, 1244 (C=C), 1179 (C–O), 1034, 916, 839 (C–C).

### 3.3. Synthesis of 4-(((Benzo[d][1,3]dioxol-5-ylmethyl)amino)(4-benzyl-5-morpholinooxazol-2-yl)methyl)benzonitile ***14***

The 4-formylbenzonitrile (1.0 mmol, 1.0 equiv.), 1,3-benzodioxole-5-methylamine (1.0 mmol, 1.0 equiv.) and sodium sulfate anhydrous (1.0 mmol, 1.0 equiv.) were placed in a sealed CEM Discover microware reaction tube (10 mL) and diluted in anhydrous toluene (1.5 mL). The mixture was stirred and heated using microwave irradiation (90 °C, 150 W) for 30 min, and ytterbium (III) triflate (0.08 equiv.) was added. The mixture was stirred and heated using microwave irradiation (60 °C, 60 W) for 5 min, and then 2-isocyano-1-morpholino-3-phenylpropan-1-one (1.2 mmol, 1.2 equiv.) was added. The new mixture was stirred and again heated using microwave irradiation (70 °C, 150 W) for 25 min, time it took to complete the reaction. Then, the solvent was evaporated under reduced pressure and the reaction crude was diluted with ethyl acetate. A Na_2_CO_3(aq.)_ (0.4 M) solution was added, and liquid–liquid extractions were carried out three times. The organic phases were collected and washed with brine three times (3 × 10 mL). The organic phase was dried with anhydrous MgSO_4_ and concentrated to dryness under vacuum. The resulting crude was purified by column chromatography using a mixture of hexanes: ethyl acetate 3:1 (*v*/*v*) as eluent to obtain a slightly yellowish oil (457.72 mg, 90% yield), R*_f_* = 0.36 (Hex-EtOAc = 3:2 *v*/*v*); ^1^H NMR (500 MHz, CDCl_3_): δ 7.62 (d, 2H, H-33, H-35, *J* = 8.6 Hz), 7.54 (d, 2H, H-32, H-36, *J* = 8.6 Hz), 7.29–7.25 (m, 2H, H-27, H-29), 7.23–7.17 (m, 3H, H-28, H-26, H-30), 6.80 (dd, 1H, H-16, *J* = 1.7, 0.5 Hz), 6.71 (dd, 1H, H-22, *J* = 7.9, 0.5 Hz), 6.67 (dd, 1H, H-23, *J* = 7.9, 1.7 Hz), 5.91 (s, 2H, H-19), 4.88 (s, 1H, H-12), 3.81 (s, 1H, H-24), 3.70–3.67 (m, 4H, H-2, H-6), 3.65–3.61 (m, 2H, H-14), 2.92–2.89 (m, 4H, H-3, H-5) ppm; ^13^C NMR (125 MHz, CDCl_3_): δ 157.5 (C-9), 152.3 (C-7), 147.7 (C-17), 146.7 (C-21), 144.6 (C-31), 139.2 (C-25), 132.9 (C-15), 132.3 (C-33, C-35), 128.3 (C-26, C-30, C-27, C-29, C-32, C-36), 126.1 (C-28), 124.7 (C-37), 121.4 (C-22), 188.5 (C-11), 111.7 (C-31), 108.7 (C-16), 108.0 (C-23), 100.8 (C-19), 66.6 (C-2, C-6), 59.4 (C-12), 51.2 (C-14), 50.8 (C-3, C-5), 31.7 (C-24) ppm; HRMS: (ESI^+^) *m/z* calcd. for [M − H]^+^ C_30_H_29_N_4_O_4_^+^ 509.2183, found 509.2188 (error = 1.0 ppm); IR ($\nu ,cm^{-1}$): 3324 (aryl H), 2958, 2892, 2854 (C–H), 1630, 1607 (N–H), 2227 (C≡N), 1441 (C=N), 1374, 1298 (C=C), 1120 (C–O–C), 1032, 992, 920, 799, 732 (C–C).

### 3.4. Synthesis One Pot of 4-(6-(Benzo[d][1,3]dioxol-5-ylmethyl)-2-benzyl-3-morpholino-5-oxo-6,7-dihydro-5H-pyrrolo[3,4-b]pyridin-7-yl)benzonitrile ***17***

General Procedure (GP): 4-formylbenzonitrile (1.0 mmol, 1.0 equiv.), 1,3-benzodioxole-5-methylamine (1.0 mmol, 1.0 equiv.) and sodium sulfate anhydrous (1.0 mmol, 1.0 equiv.) were placed in a sealed CEM Discover microwave reaction tube (10 mL) and diluted in anhydrous toluene (1.5 mL). The mixture was stirred and heated using microwave irradiation (90 °C, 150 W) for 30 min, and ytterbium (III) triflate (0.08 equiv.) was added. The mixture was stirred and heated using microwave irradiation (60 °C, 60 W) for 5 min, and then 2-isocyano-1-morpholino-3-phenylpropan-1-one (1.2 mmol, 1.2 equiv.) was added. The new mixture was stirred and again heated using microwave irradiation (70 °C, 150 W) for 25 min, and then maleic anhydride (1.3 mmol, 1.0 equiv.) was added. Finally, the reaction mixture was stirred and heated using microwave irradiation (80 °C, 150 W) for 20 min. Then, the solvent was removed to dryness under vacuum. The crude was extracted using ethyl acetate (3 × 10.0 mL) and Na_2_CO_3(aq.)_ (3 × 10.0 mL), and then washed with brine (3 × 10 mL). The organic layer was dried using anhydrous MgSO_4_, filtered, and concentrated to dryness under vacuum. The new crude was purified by silica-gel column chromatography using mixtures of hexanes (Hex) and ethyl acetate (EtOAc) in 4:1 to 3:2 (*v*/*v*) proportions as mobile phase to isolate the corresponding pyrrolo[3,4-*b*]pyridin-5-one-7-yl benzonitrile (490.15 mg, 90% yield) as yellow oil, R*_f_* = 0.30 (Hex-EtOAc = 3:2 *v*/*v*); ^1^H NMR (500 MHz, CDCl_3_): δ 7.90 (s, 1H, H-15), 7.67–7.64 (m, 2H, H-25, H-27), 7.28–7.25 (m, 2H, H-24, H-28), 7.18–7.10 (m, 5H, H-18, H-22), 6.70 (dd, 1H, H-37, *J* = 7.9, 0.4 Hz), 6.67 (d, 1H, H-31, *J* = 1.7 Hz), 6.57 (dd, 1H, H-38, *J* = 7.9, 1.7 Hz), 5.92 (d, 1H, H-34, *J* = 1.4 Hz), 5.91 (d, 1H, H-34’, *J* = 1.4 Hz), 5.33 (d, 1H, H-29, *J* = 14.5 Hz), 5.29 (s, 1H, H-11), 4.26 (d, 1H, H-16, *J* = 14.0 Hz), 4.15 (d, 1H, H-16’, *J* = 14.0 Hz), 3.81 (t, 4H, H-2, H-6, *J* = 4.6 Hz), 3.73 (d, 1H, H-29’, *J* = 4.6 Hz), 2.87–2.80 (m, 4H, H-3, H-5) ppm; ^13^C NMR (125 MHz, CDCl_3_): δ 167.0 (C-13), 162.3 (C-8), 159.2 (C-10), 148.2 (C-7), 148.1 (C-32), 147.3 (C-36), 141.1 (C-23), 139.0 (C-17), 132.7 (C-25, C-27), 130.0 (C-30), 128.7 (C-18, C-22), 128.6 (C-24, C-28), 128.2 (C-19, C-21), 126.2 (C-20), 123.9 (C-15), 123.5 (C-14), 121.8 (C-38), 118.3 (C-40), 112.5 (C-26), 108.7 (C-31), 108.3 (C-37), 101.2 (C-34), 67.0 (C-2, C-6), 63.7 (C-11), 53.0 (C-3, C-5), 44.0 (C-29), 39.9 (C-16) ppm; HRMS: (ESI^+^) *m/z* calcd. for [M − H]^+^ C_33_H_29_N_4_O_4_^+^ 545.2183, found 545.2192 (error = 1.6 ppm); IR ($\nu ,cm^{-1}$): 3419 (aryl H), 3005, 2968 (C–H), 1710 (C=O, ketone), 1490 (C=N), 1441 (C=C), 1416 (C–O–C), 1361, 1220, 1117, 1034, 905 (C–C).

### 3.5. Synthesis of 7-(4-(1H-Tetrazol-5-yl)phenyl)-6-(benzo[d][1,3]dioxol-5-ylmethyl)-2-benzyl-3-morpholino-6,7-dihydro-5H-pyrrolo[3,4-b]pyridin-5-one ***19***

According to the GP, compound **17** was synthesized, and once the reaction was finished, the solvent was evaporated to dryness, and the crude was transferred to a Pyrex pressure tube (5 mL), sodium azide (1.5 mmol, 1.5 equiv.) and zinc bromide (2.0 mmol, 2.0 equiv.) were added. The mixture was dissolved in 1.5 mL of H_2_O and 1.5 mL of isopropyl alcohol, and then the mixture of reaction was heated in an oil bath at 120 °C for 40 hours. Subsequently, the reaction mixture was allowed to cool to room temperature, and a mixture 1:1 *v*/*v* of hexanes with ethyl acetate was added at 0 °C, stirred for 10 min, and then filtered and washed with hot water and hot ethyl acetate. The resulting solid was dried under high vacuum overnight, obtaining 264.44 mg of a light brown solid (45% yield); mp > 250 °C; ^1^H NMR (500 MHz, DMSO-*d_6_*): δ 7.96 (d, 2H, H-25, H-27, *J* = 7.9 Hz), 7.92 (s, 1H, H-15), 7.16 (d, 2H, H-24, H-28, *J* = 7.9 Hz), 7.14–7.05 (m, 5H, H-18, H-22), 6.79 (d, 1H, H-37, *J* = 7.9 Hz), 6.72 (d, 1H, H-31, *J* = 1.7 Hz), 6.62–6.60 (m, 1H, H-38), 5.95 (d, 1H, H-34, *J* = 1.0 Hz), 5.93 (d, 1H, H-34’, *J* = 1.0 Hz), 5.44 (s, 1H, H-11), 5.00 (d, 1H, H-29, *J* = 15.1 Hz), 4.28 (d, 1H, H-16, *J* = 14.2 Hz), 4.06 (d, 1H, H-16’, *J* = 14.2 Hz), 3.76 (d, 1H, H-29’, *J* = 15.1 Hz), 3.72–3.66 (m, 4H, H-2, H-6), 2.89–2.76 (m, 4H, H-3, H-5) ppm; ^13^C NMR (125 MHz, DMSO-*d_6_*): δ 166.5 (C-13), 161.7 (C-8), 160.2 (C-10), 148.1 (C-7), 147.9 (C-32), 146.9 (C-36), 139.7 (C-17), 136.5 (C-26), 131.1 (C-30), 130.1 (C-40), 129.0 (C-18, C-22), 128.7 (C-24, C-28), 128.5 (C-19, C-21), 127.5 (C-25, C-27), 126.3 (C-20), 124.0 (C-15), 123.8 (C-14), 121.7 (C-38), 108.7 (C-31), 108.6 (C-37), 101.3 (C-34), 66.8 (C-2, C-6), 64.1 (C-11), 52.8 (C-3, C-5), 43.7 (C-29), 40.4 (C-16) ppm; HRMS: (ESI^+^) *m/z* calcd. for [M − H]^+^ C_33_H_30_N_7_O_4_^+^ 588.2354, found 588.2348 (error = 1.0 ppm); IR ($\nu ,cm^{-1})$: 2973, 2867 (C–H), 2368, 2187, 2156, 2090 (C=N), 1993, 1922, 1741 (C=C), 1701 (C=O, ketone), 1599, 1493, 1445, 1397, 1365 (C=N), 1290, 1246, 1220 (C–O–C), 1158, 1114, 1034, 1013, 915, 862, 796, 787, 729, 703, 650, 623 (C–C).

### 3.6. Synthesis of 6-(Benzo[d][1,3]dioxol-5-ylmethyl)-2-benzyl-7-(4-(4,6-diamino-1,3,5-triazin-2-yl)phenyl)-3-morpholino-6,7-dihydro-5H-pyrrolo[3,4-b]pyridin-5-one ***21***

According to the GP, compound **17** was synthesized, and once the reaction was finished, the solvent was evaporated to dryness, and to the crude, 1.5 mL of 2-methoxyethan-1-ol, dicyandiamide (1.2 mmol, 1.2 equiv.), and potassium hydroxide (0.008 mmol, 0.0008 equiv.) were added. The mixture was stirred and heated using microwave irradiation (150 °C, 250 W) for 90 min. Subsequently, the reaction mixture was allowed to cool to room temperature. Then, hot water was stirred for 10 min, and then filtered and washed with hot water and hot ethyl acetate. The resulting solid was dried under a high vacuum overnight, obtaining 408.65 mg of a yellow solid (65% yield); mp > 250 °C; ^1^H NMR (500 MHz, CDCl_3_): δ 8.28 (d, 2H, H-25, H-27, *J* = 8.6 Hz), 7.92 (s, 1H, H-11), 7.20 (d, 2H, H-24, H-28, *J* = 8.6 Hz), 7.15–7.09 (m, 5H, H-18, H-22), 6.70 (d, 1H, H-37, *J* = 7.9 Hz), 6.69 ( d, 1H, H-31, *J* = 1.7 Hz), 6.59 (dd, 1H, H-38, *J* = 7.8, 1.7 Hz), 5.92 (d, 1H, H-34, *J* = 1.4 Hz), 5.91 (d, 1H, H-34’, *J* = 1.4 Hz), 5.47 (bs, 4H, H-46, H-47), 5.33 (d, 1H, H-29, *J* = 14.8 Hz), 5.30 (s, 1H, H-11), 4.80 (d, 1H, H-16, *J* = 13.9 Hz), 4.12 (d, 1H, H-16’, *J* = 13.9 Hz), 3.81–3.77 (m, 4H, H-2, H-6), 3.66 (d, 1H, H-29’, *J* = 14.8 Hz), 2.86–2.77 (m, 4H, H-3, H-5) ppm; ^13^C NMR (125 MHz, CDCl_3_): δ 171.8 (C-40), 167.6 (C-42, C-44), 167.0 (C-13), 162.2 (C-8), 160.2 (C-10), 148.0 (C-17), 147.9 (C-32), 147.2 (C-36), 139.2 (C-7), 138.9 (C-23), 137.0 (C-26), 130.4 (C-30), 129.0 (C-25, C-27), 128.7 (C-18, C-22), 128.2 (C-24, C-28), 128.0 (C-19, C-21), 126.1 (C-20), 124.0 (C-15), 123.8 (C-14), 121.9 (C-38), 108.9 (C-37), 108.3 (C-31), 101.1 (C-34), 67.1 (C-2, C-6), 64.1 (C-11), 53.0 (C-3, C-5), 43.7 (C-29), 40.0 (C-16) ppm; HRMS: (ESI^+^) *m/z* calcd. for [M − H]^+^ C_35_H_33_N_8_O_4_^+^ 629.2619, found 629.2647 (error = 4.4 ppm); IR ($\nu ,cm^{-1}$): 3419 (aryl H), 3009, 2964 (C–H), 1715 (C=O, ketone), 1609, 1542 (N–H, secondary amine), 1485, 1440 (C=N), 1419, 1357 (C=C), 1220 (C–O–C), 1096, 1046, 902, 818, 778, 695.

## 4. Conclusions

A couple of new polyheterocycles were synthesized via synthetic strategies that involve combinations of MCRs with cascade processes and click reactions. Such target compounds were designed and synthesized using 4-formylbenzonitrile as a pivotal *b*i-functionalized orthogonal reagent. The syntheses were accomplished with good chemical yields, high atom economy, and outstanding structural complexity both stepwise and in one pot manner. It is worth highlighting the utility of the Ugi-Zhu reaction as a structural generation tool, in turn allowing the control over several points of diversity: varying the functional groups (appendage diversity), and the generation of different molecular skeletons (diversity skeletal) through post-functionalization processes. In line with the diversity-oriented synthesis (DOS) approach, both polyheterocyclic compounds correspond to a reagent-based approach because the new, structurally diverse polyheterocycles were synthesized from common scaffolds, first the 5-aminooxazole and then the pyrrolo[3,4-*b*]pyridin-5-one.

## Data Availability

Not applicable.

## Supplementary Materials

The following supporting information can be downloaded at: https://www.mdpi.com/article/10.3390/molecules28104087/s1. Synthesis of the imine **12** (Table S1). Characterization of the imine **12** (Figures S1–S4). Synthesis of the 5-aminooxazole **14** (Table S2). Characterization of the 5-aminooxazole **14** (Figures S5–S8). Synthesis of the cyano-pyrrolo[3,4-*b*]pyridin-5-one **17** (Table S3). Characterization of the cyano-pyrrolo[3,4-*b*]pyridin-5-one **17** (Figures S9–S12). Synthesis of the tetrazolyl-pyrrolo[3,4-*b*]pyridin-5-one **19** (Table S4). Characterization of the tetrazolyl-pyrrolo[3,4-*b*]pyridin-5-one **19** (Figures S13–S22). Synthesis of the triazine-pyrrolo[3,4-*b*]pyridin-5-one **21** (Table S5). Characterization of the triazine-pyrrolo[3,4-*b*]pyridin-5-one **21** (Figures S23–S32).

## Sample Availability

Samples of the compounds are available from the authors upon request.
