# Supplementary material for: Synthesis of New Polyheterocyclic Pyrrolo[3,4-b]pyridin-5-ones via an Ugi-Zhu/Cascade/Click Strategy
Source: Molecules. 2023 May 14;28(10):4087. doi: 10.3390/molecules28104087 (PMC10221234; doi:10.3390/molecules28104087)
Supplement: Supplementary file 1 [file molecules-28-04087-s001.zip › molecules-2385315-supplementary.pdf]

SUPPLEMENTARY MATERIALS

Synthesis of new polyheterocyclic pyrrolo[3,4-*b*]pyridin-5-ones via an Ugi-Zhu / cascade / click strategy

Roberto E. Blanco-Carapia [1], Enrique A. Aguilar-Rangel [1], Mónica A. Rincón-Guevara [2], Alejandro Islas-Jácome [1,*], and Eduardo González-Zamora [1,*]

* Correspondence: aij@xanum.uam.mx (A.I.-J); egz@xanum.uam.mx (E.G.-Z.)

TABLE OF CONTENTS

Spectra of the compounds	Page
Synthesis of the imine **12**…………………………………………………………………………	S2
^{1}H-NMR and ^{13}C-NMR of the imine **12**………………………………………………………	S3
HRMS and FT-IR of the imine **12**………………………………………………………………..	S4
Synthesis of the 5-aminooxazole **14**……………………………………………………………..	S5
^{1}H-NMR and ^{13}C-NMR of the 5-aminooxazole **14**………………………………………………..	S6
HRMS and FT-IR of the 5-aminooxazole **14**……………………………………………………….	S7
Synthesis of the cyano-pyrrolo[3,4-*b*]pyridin-5-one **17**……………………………………...…	S8
^{1}H-NMR and ^{13}C-NMR of the cyano-pyrrolo[3,4-*b*]pyridin-5-one **17**…………………………...	S9
HRMS and FT-IR of the cyano-pyrrolo[3,4-*b*]pyridin-5-one **17**….…………………………………	S10
Synthesis of the tetrazolyl-pyrrolo[3,4-*b*]pyridin-5-one **19**……….……….……………………..	S11
^{1}H-NMR and ^{13}C-NMR of the tetrazolyl-pyrrolo[3,4-*b*]pyridin-5-one **19**……….……….……...	S12
2D-NMR (COSY and HSQC-part I) of the tetrazolyl-pyrrolo[3,4-*b*]pyridin-5-one **19**…..…….....	S13
2D-NMR (HSQC-part II and HMBC-part I) of the tetrazolyl-pyrrolo[3,4-*b*]pyridin-5-one **19**….	S14
2D-NMR (HMBC-part II and HMBC-part III) of the tetrazolyl-pyrrolo[3,4-*b*]pyridin-5-one **19**..	S15
HRMS and FT-IR of the tetrazolyl-pyrrolo[3,4-*b*]pyridin-5-one **19**……………………………….	S16
Synthesis of the triazine-pyrrolo[3,4-*b*]pyridin-5-one **21**……………………………………………	S17
^{1}H-NMR and ^{13}C-NMR of the triazine-pyrrolo[3,4-*b*]pyridin-5-one **21**……………………………	S18
2D-NMR (COSY and HSQC-part I) of the triazine-pyrrolo[3,4-*b*]pyridin-5-one **21**……………...	S19
2D-NMR (HSQC-part II and HMBC-part I) of the triazine-pyrrolo[3,4-*b*]pyridin-5-one **21**…….	S20
2D-NMR (HMBC-part II and HMBC-part III) of the triazine-pyrrolo[3,4-*b*]pyridin-5-one **21**….	S21
HRMS and FT-IR of the triazine-pyrrolo[3,4-*b*]pyridin-5-one **21**…………………………………..	S22

Table S1. Synthesis of the Schiff base **12**.

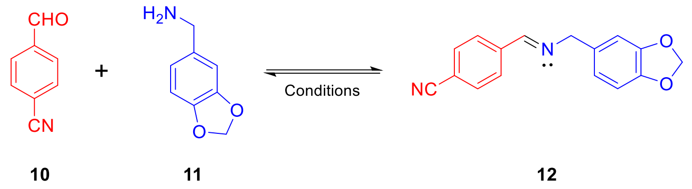

Entry[a]	Solvent	Additive	Temperature[b] (°C)	Time (h)	Yield[c] (%)
1	MeOH	--	rt	24	--
2	MeOH	--	50	1	--
3	PhMe	--	70	1	traces
4	PhMe	--	80	1	35
5	PhMe	--	90	0.5	69
6	PhMe	molecular sieves	90	0.5	73
7[d]	PhMe	Na_2SO_4	90	0.5	90

[a] Concentration in all inputs was [2 M]. [b] MW were the heating source (except for entry 1).
[c] Calculated after purification by precipitation–filtration. [d] Optimal reaction conditions.

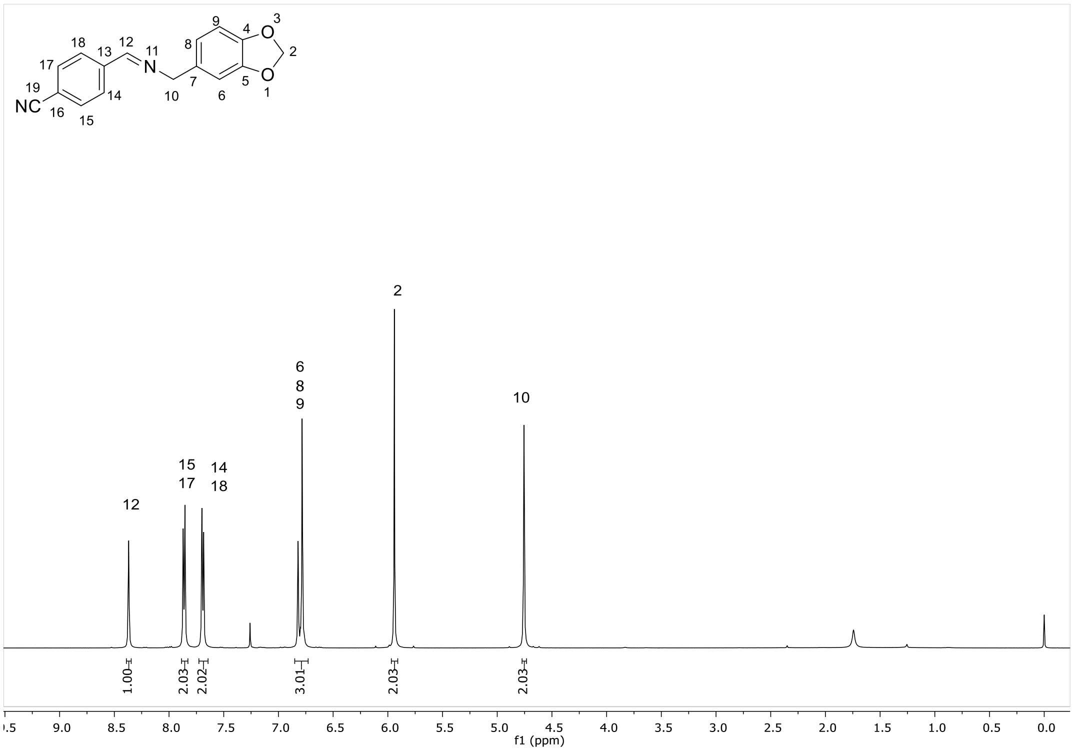

Figure S1. ^{1}H-NMR (500 MHz, $CDCl_3$) spectrum of the imine **12**

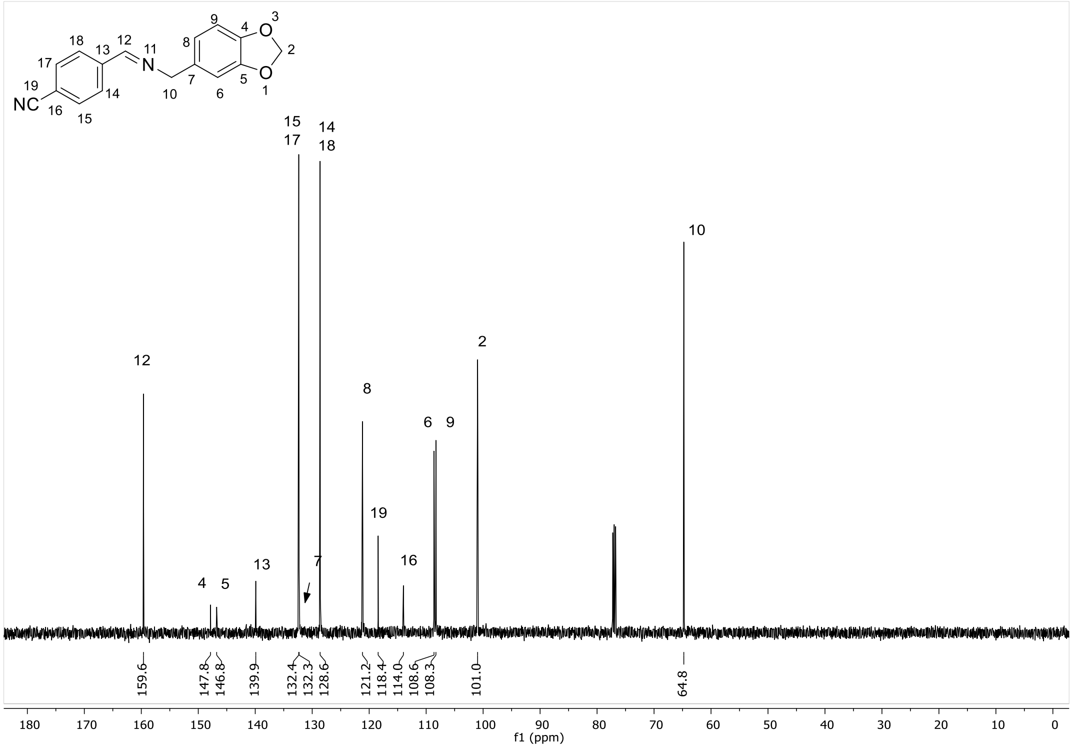

Figure S2. ^{13}C-NMR (125 MHz, $CDCl_3$) spectrum of the imine **12**

Mass Spectrum SmartFormula Report

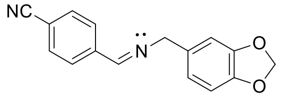

Chemical Formula: $C_{16}H_{12}N_2O_2$
Exact Mass: 264.09

Acquisition Parameter

Source Type	ESI	Ion Polarity	Positive	Set Nebulizer	0.3 Bar
Focus	Active			Set Dry Heater	180 °C
Scan Begin	50 m/z	Set Capillary	4500 V	Set Dry Gas	4.0 l/min
Scan End	3000 m/z	Set End Plate Offset	-500 V	Set Divert Valve	Waste

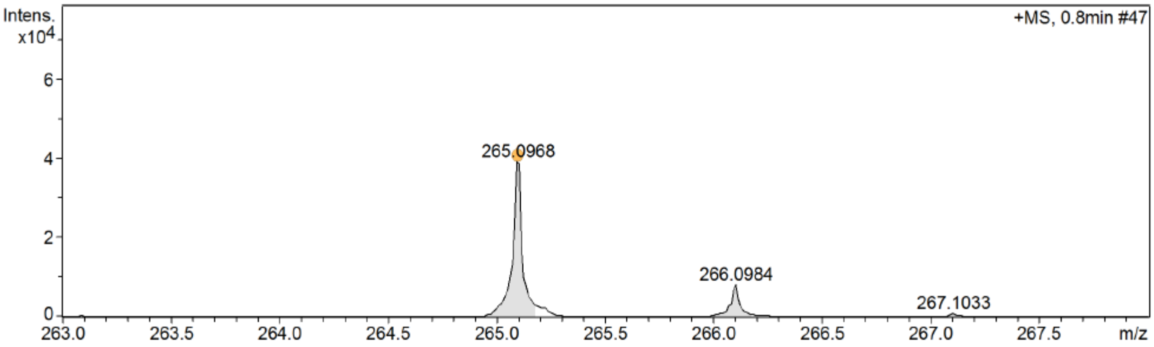

Meas. m/z	#	Ion Formula	Sum Formula	m/z	err [ppm]	err [mDa]	mSigma	# Sigma
265.0968	1	C16H13N2O2	C16 H12 N2 O2	265.0972	-1.3	-0.3	14.2	1

Figure S3. HRMS (ESI⁺-TOF) spectrum of the imine **12**

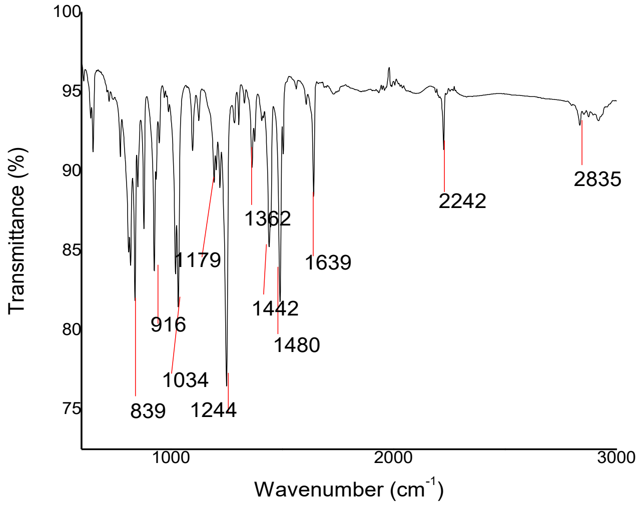

Figure S4. FT-IR (ATR) spectrum of the imine **12**

Table S2. Synthesis of the 5-aminooxazole **14**.

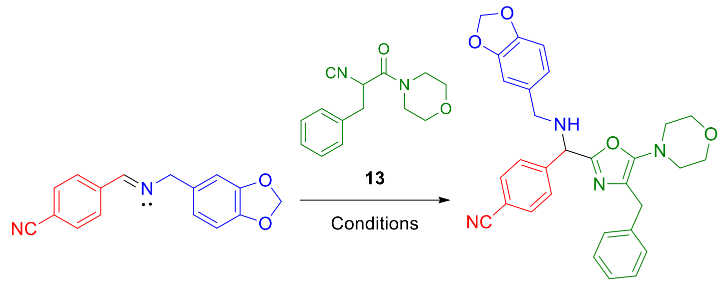

Entry[a]	Catalyst	Temperature[b] (°C)	Time (h)	Yield[c] (%)
1	--	rt	24	--
2	--	50	1.5	--
3	--	60	1.5	traces
4	--	70	1.5	traces
5	--	80	1.5	traces
6	$Sc(OTf)_3$ (3% mol)	60	1.5	26
7	$Sc(OTf)_3$ (3% mol)	70	1.5	39
8	$Sc(OTf)_3$ (5% mol)	70	1	48
9	$Sc(OTf)_3$ (8% mol)	70	1	49
10	$InCl_3$ (5% mol)	70	1	28
11	$AlCl_3$ (5% mol)	70	1	7
12	$Yb(OTf)_3$ (5% mol)	70	0.5	87
13[d]	$Yb(OTf)_3$ (8% mol)	70	0.5	90

[a] PhMe was the solvent, and concentration in all inputs was [1.5 M]. [b] MW were the heating source (except for entry 1). [c] Calculated after purification by preparative TLC. [d] Optimal reaction conditions.

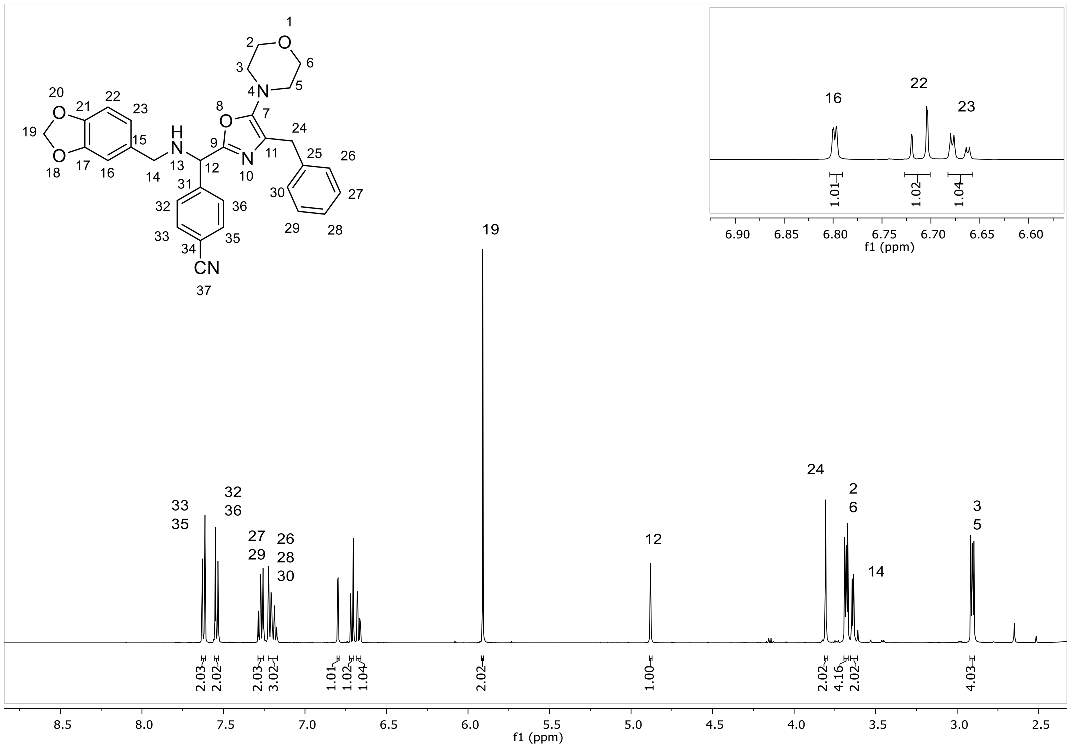

Figure S5. ^{1}H-NMR (500 MHz, $CDCl_3$) spectrum of the 5-aminooxazole **14**

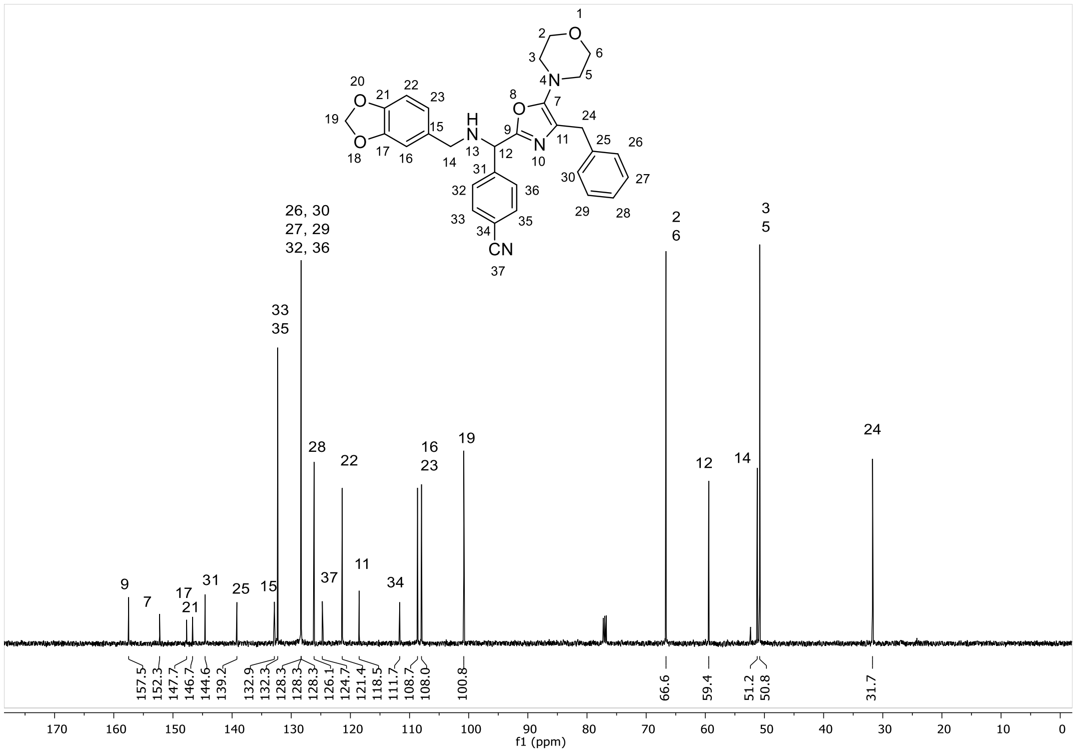

Figure S6. ^{13}C-NMR (125 MHz, $CDCl_3$) spectrum of the 5-aminooxazole **14**

Mass Spectrum SmartFormula Report

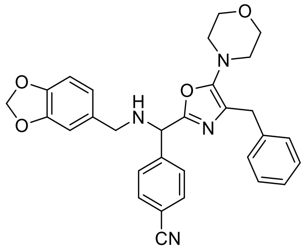

Chemical Formula: $C_{30}H_{28}N_4O_4$
Exact Mass: 508.21

Acquisition Parameter

Source Type	ESI	Ion Polarity	Positive			Set Nebulizer	0.3 Bar
Focus	Active					Set Dry Heater	180 °C
Scan Begin	50 m/z	Set Capillary	4500 V			Set Dry Gas	4.0 l/min
Scan End	3000 m/z	Set End Plate Offset	-500 V			Set Divert Valve	Waste

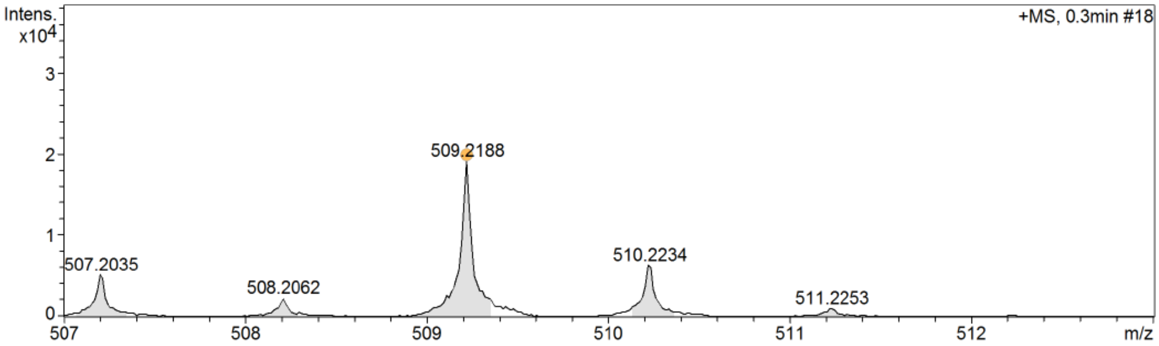

Meas. m/z	#	Ion Formula	Sum Formula	m/z	err [ppm]	|err| [mDa]	mSigma	# Sigma
509.2188	1	C30H29N4O4	C30 H28 N4 O4	509.2183	-1.0	0.5	9.7	1
1017.4273	1	C60H57N8O8	C60 H57 N8 O8	1017.4294	2.0	2.1	28.4	1

Figure S7. HRMS (ESI$^+$-TOF) spectrum of the 5-aminooxazole **14**

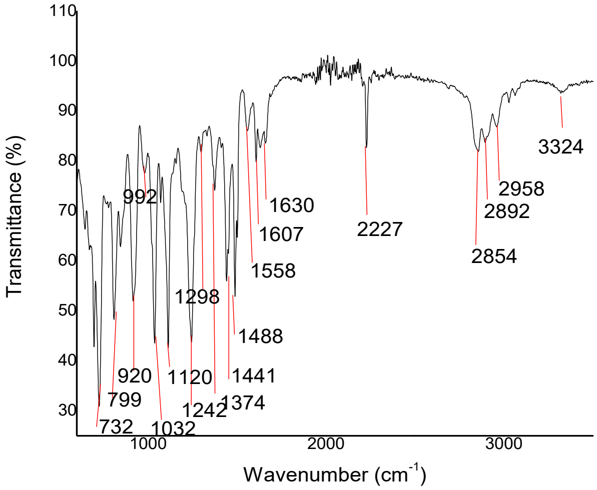

Figure S8. FT-IR (ATR) spectrum of the 5-aminooxazole **14**

Table S3. Synthesis of the cyano-pyrrolo[3,4-*b*]pyridin-5-one **17**.

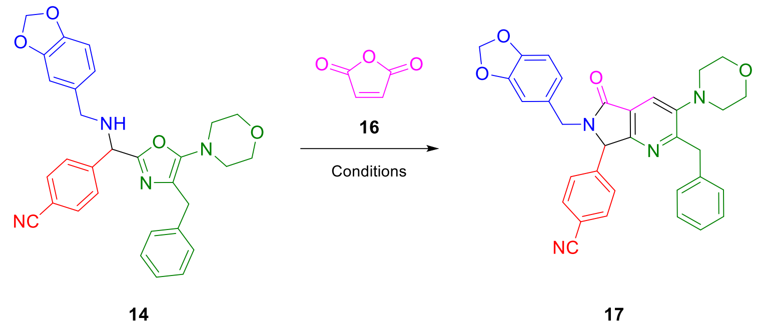

Entry[a]	Temperature[b] (°C)	Time (h)	Yield[c] (%)
1	25	1	--
2	50	1	--
3	60	1/3	31
4	70	1/3	66
5[d]	80	1/3	96

[a] PhMe was the solvent, and concentration in all inputs was [1.5 M]. [b] MW were the heating source. [c] Calculated after purification by preparative TLC. [d] Optimal reaction conditions.

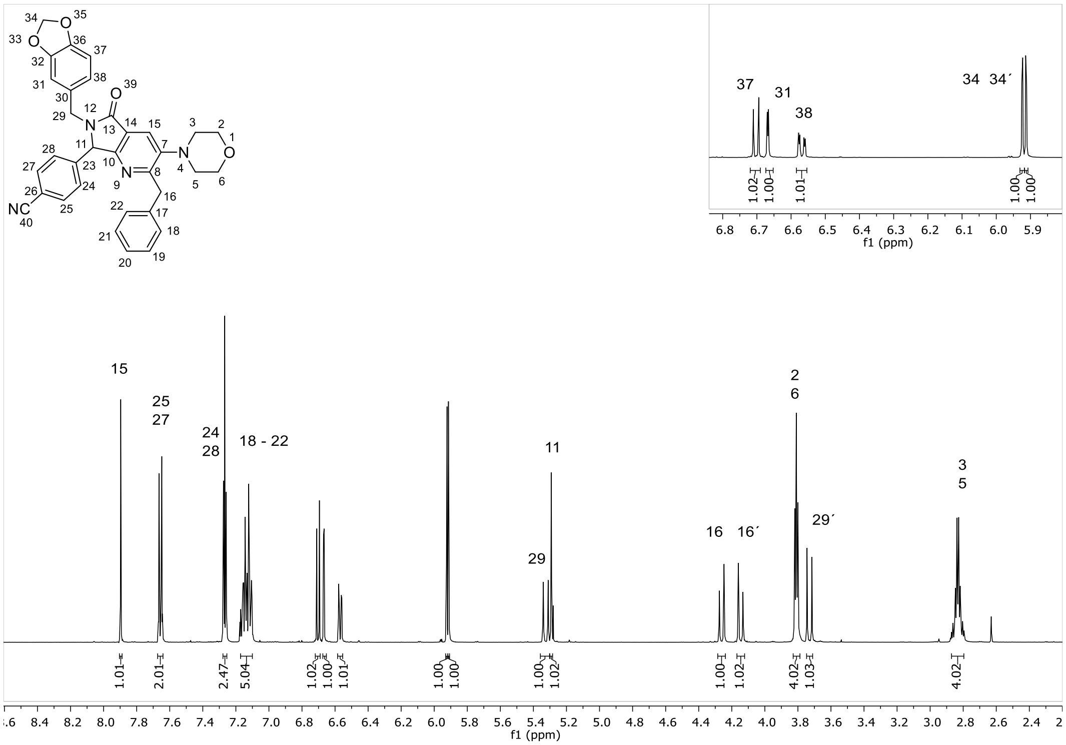

Figure S9. ^{1}H-NMR (500 MHz, $CDCl_3$) spectrum of cyano-pyrrolo[3,4-*b*]pyridin-5-one **17**

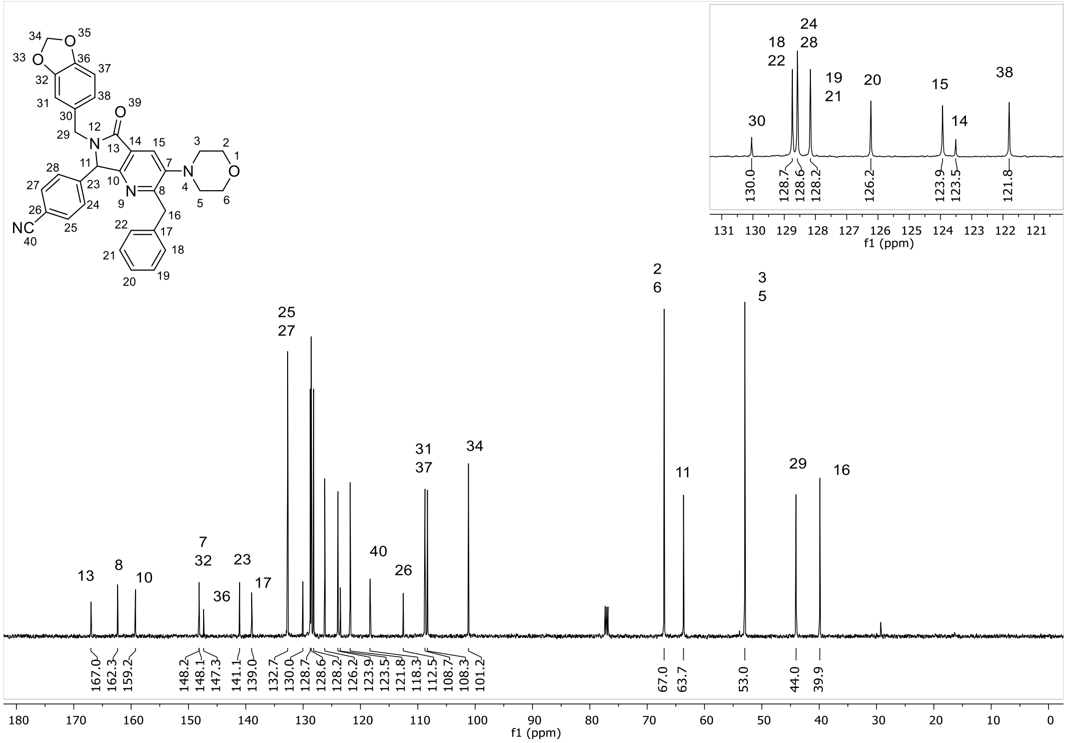

Figure S10. ^{13}C-NMR (125 MHz, $CDCl_3$) spectrum of the cyano-pyrrolo[3,4-*b*]pyridin-5-one **17**

Mass Spectrum SmartFormula Report

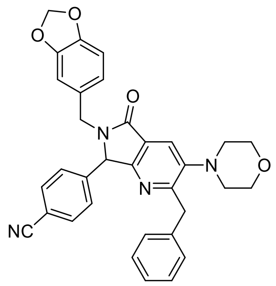

Chemical Formula: $C_{33}H_{28}N_4O_4$
Exact Mass: 544.21

Acquisition Parameter

Source Type	ESI	Ion Polarity	Positive	Set Nebulizer	0.3 Bar
Focus	Active			Set Dry Heater	180 °C
Scan Begin	50 m/z	Set Capillary	4500 V	Set Dry Gas	4.0 l/min
Scan End	3000 m/z	Set End Plate Offset	-500 V	Set Divert Valve	Waste

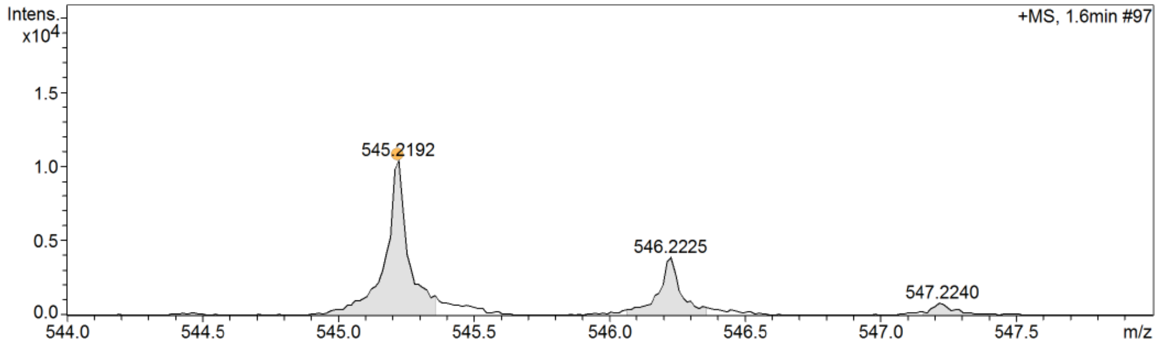

Meas. m/z	#	Ion Formula	Sum Formula	m/z	err [ppm]	err [mDa]	mSigma	# Sigma
545.2192	1	C33H29N4O4	C33 H28 N4 O4	545.2183	1.6	0.9	3.0	1

Figure S11. HRMS (ESI+-TOF) spectrum of the cyano-pyrrolo[3,4-*b*]pyridin-5-one **17**

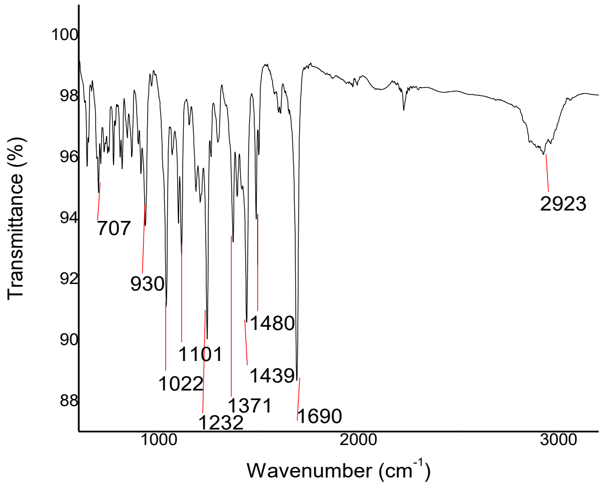

Figure S12. FT-IR (ATR) spectrum of the cyano-pyrrolo[3,4-*b*]pyridin-5-one **17**

Table S4. Synthesis of the 5-substituted-1*H*-tetrazolyl-pyrrolo[3,4-*b*]pyridin-5-one **19** via a [3+2] cycloaddition with the participation of azide anion as linear 4π-component.

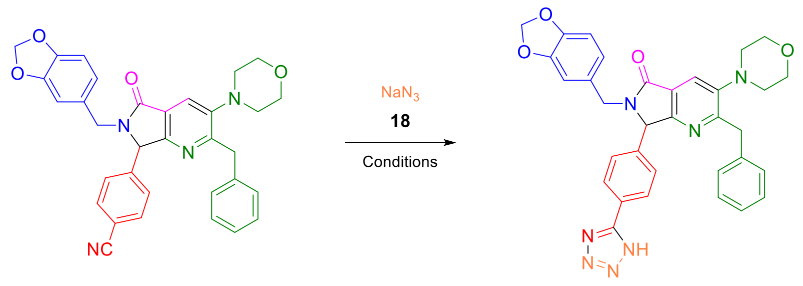

17 19

Entry[a]	Solvent	Additive	Temperature (°C)	Time (h)	Yield[b] (%)
1	DMF	Et_3NH_4Cl	120 (MW)	2	--
2	H_2O / *i*-PrOH	$ZnBr_2$	100 (reflux)	48	9
3	H_2O / *i*-PrOH	$ZnBr_2$	100 (MW)	2	6
4[c]	H_2O / *i*-PrOH	$ZnBr_2$	120 (solvothermal sealed tube)	40	45

[a] Concentration in all inputs was [2.25 M]. [b] Calculated after purification by precipitation–filtration.
[c] Optimal reaction conditions.

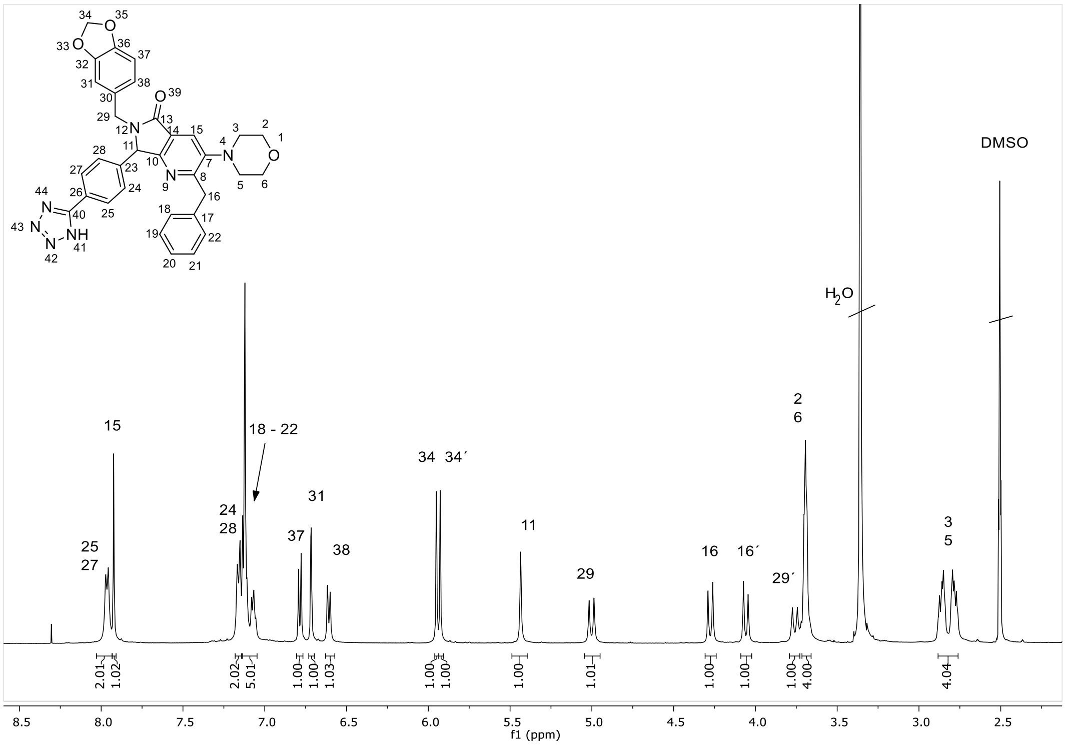

Figure S13. ^{1}H-NMR (500 MHz, d_6-DMSO) spectrum of the tetrazolyl-pyrrolo[3,4-*b*]pyridin-5-one **19**

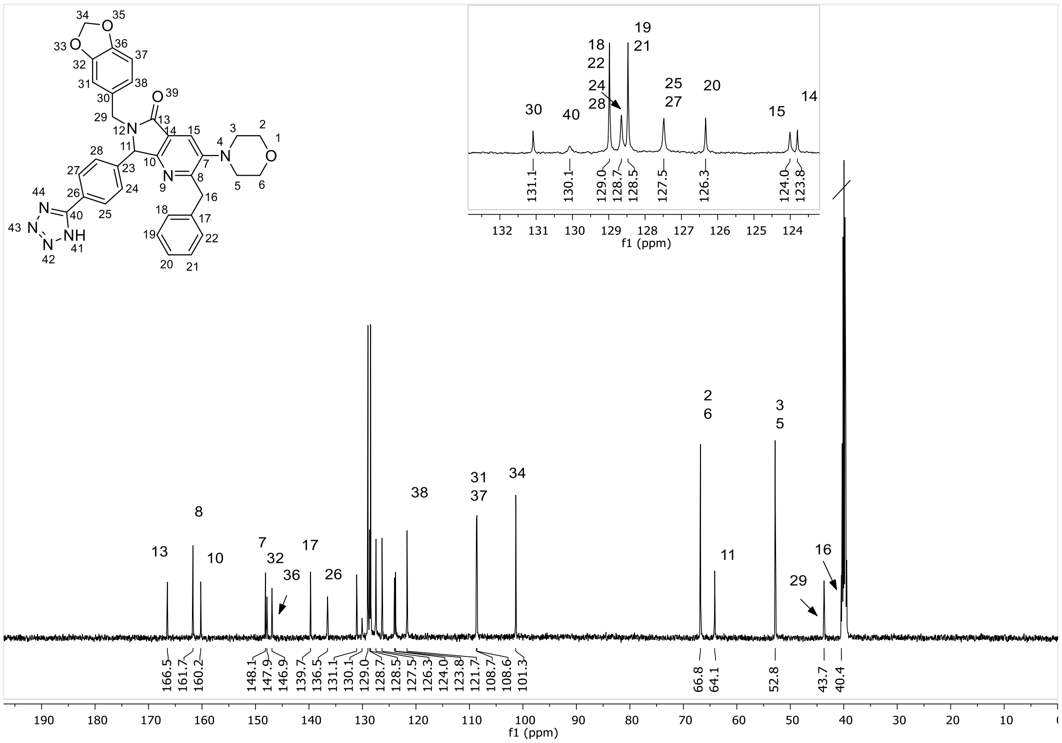

Figure S14. ^{13}C-NMR (125 MHz, d_6-DMSO) spectrum of the tetrazolyl-pyrrolo[3,4-*b*]pyridin-5-one **19**

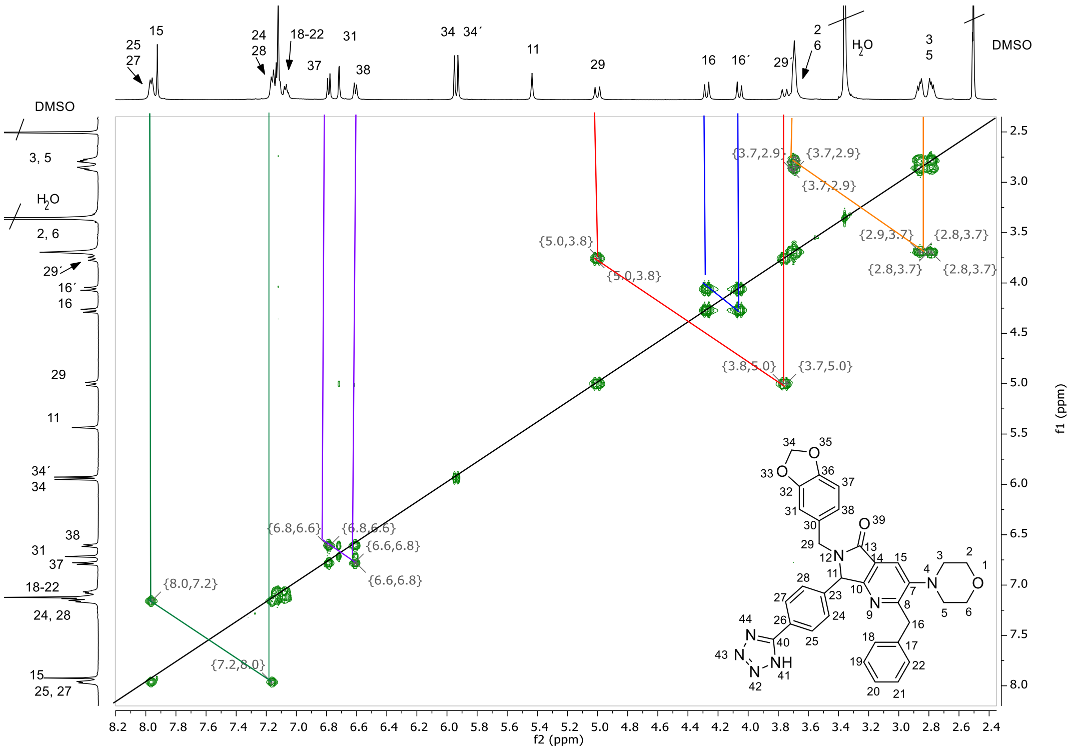

Figure S15. 2D-NMR (COSY) spectrum of the tetrazolyl-pyrrolo[3,4-*b*]pyridin-5-one **19**

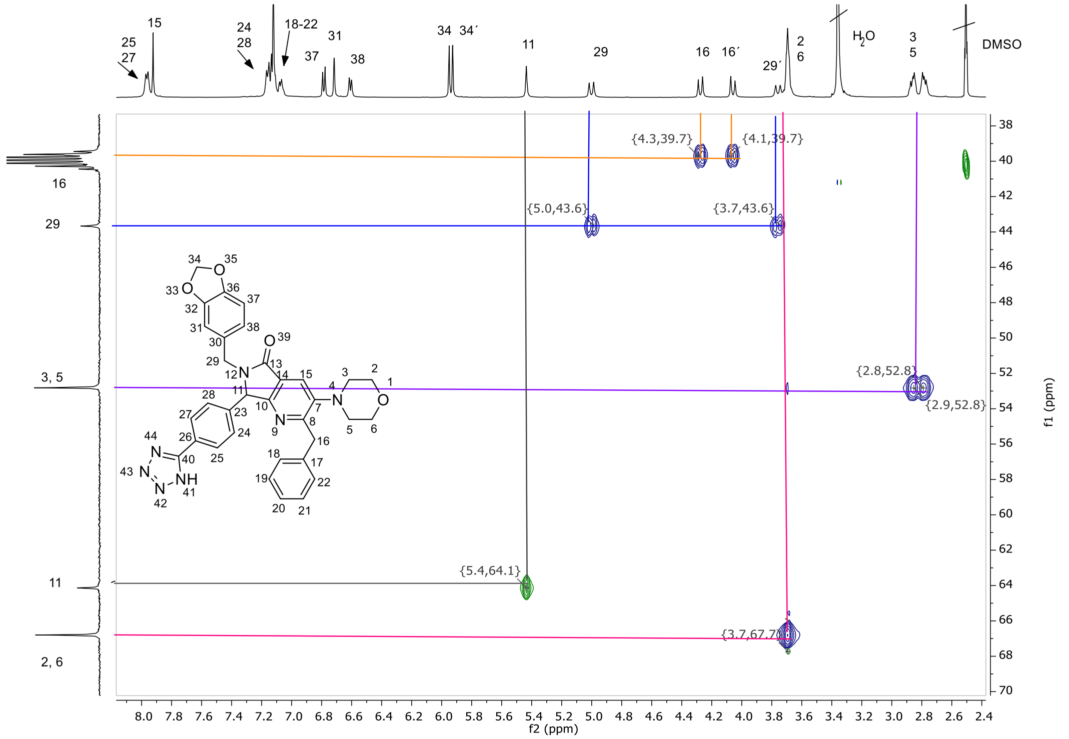

Figure S16. 2D-NMR (HSQC-part I) spectrum of the tetrazolyl-pyrrolo[3,4-*b*]pyridin-5-one **19**

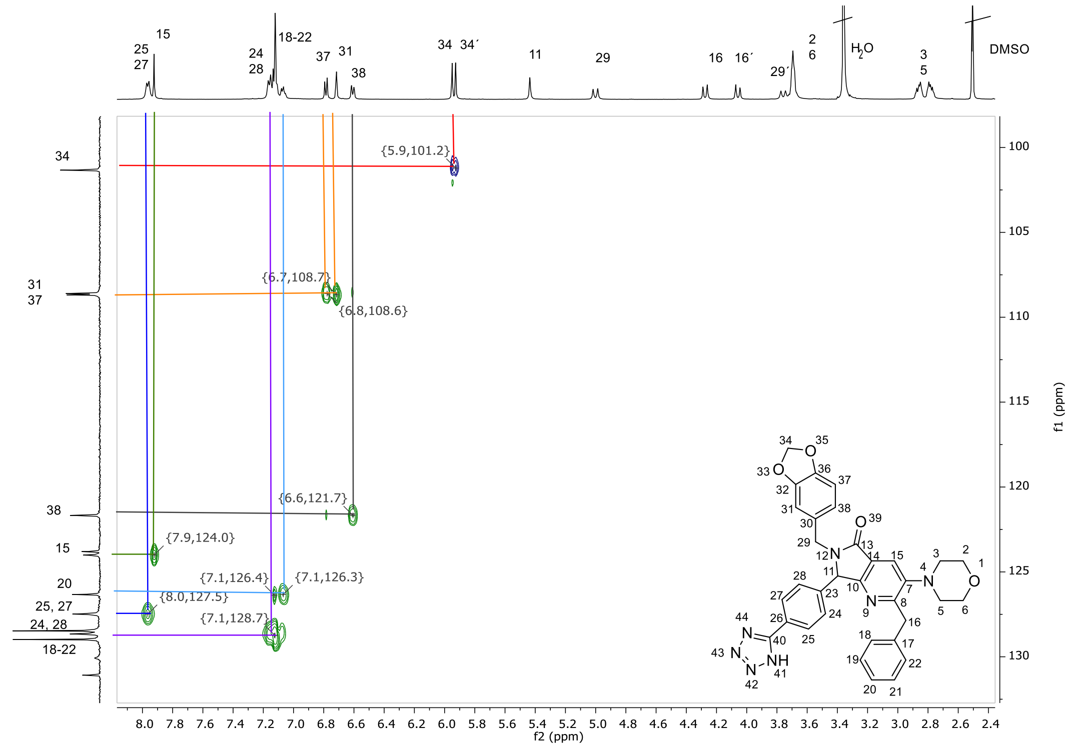

Figure S17. 2D-NMR (HSQC-part II) spectrum of the tetrazolyl-pyrrolo[3,4-*b*]pyridin-5-one **19**

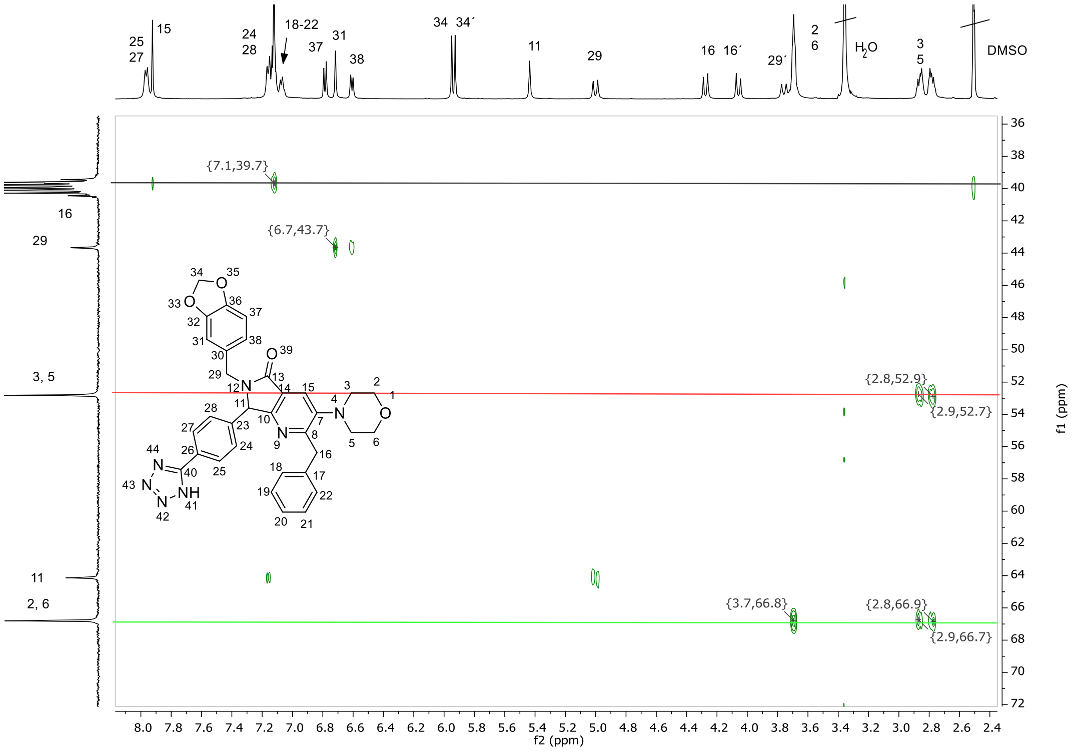

Figure S18. 2D-NMR (HMBC-part I) spectrum of the tetrazolyl-pyrrolo[3,4-*b*]pyridin-5-one **19**

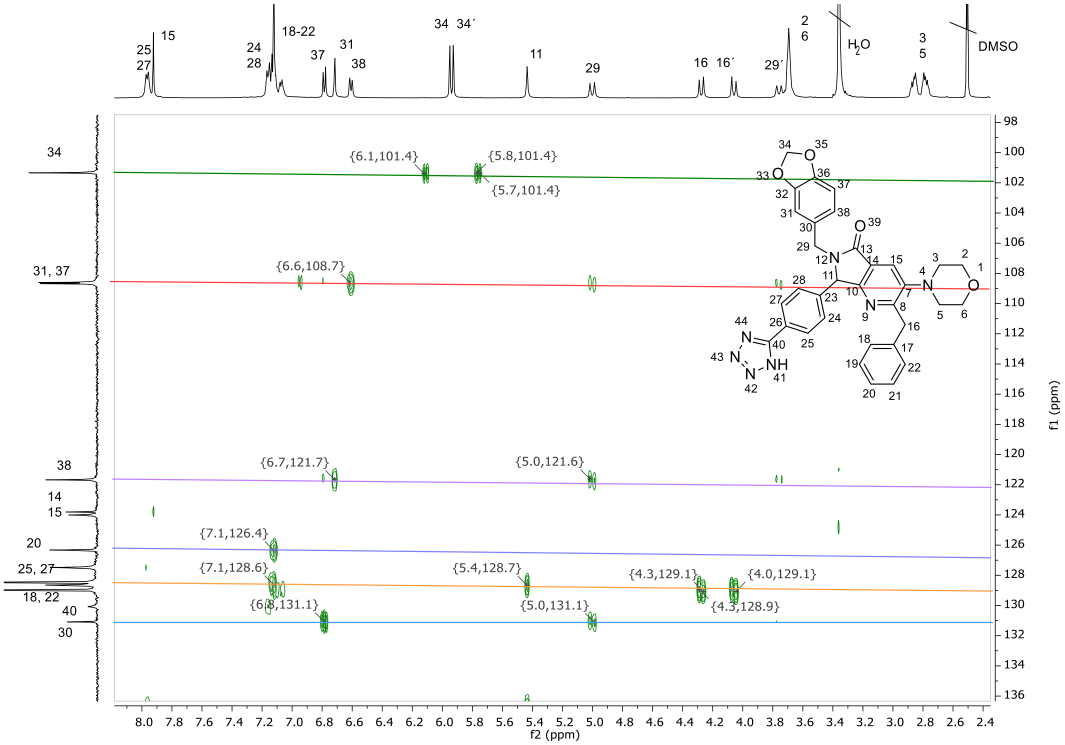

Figure S19. 2D-NMR (HMBC-part II) spectrum of the tetrazolyl-pyrrolo[3,4-*b*]pyridin-5-one **19**

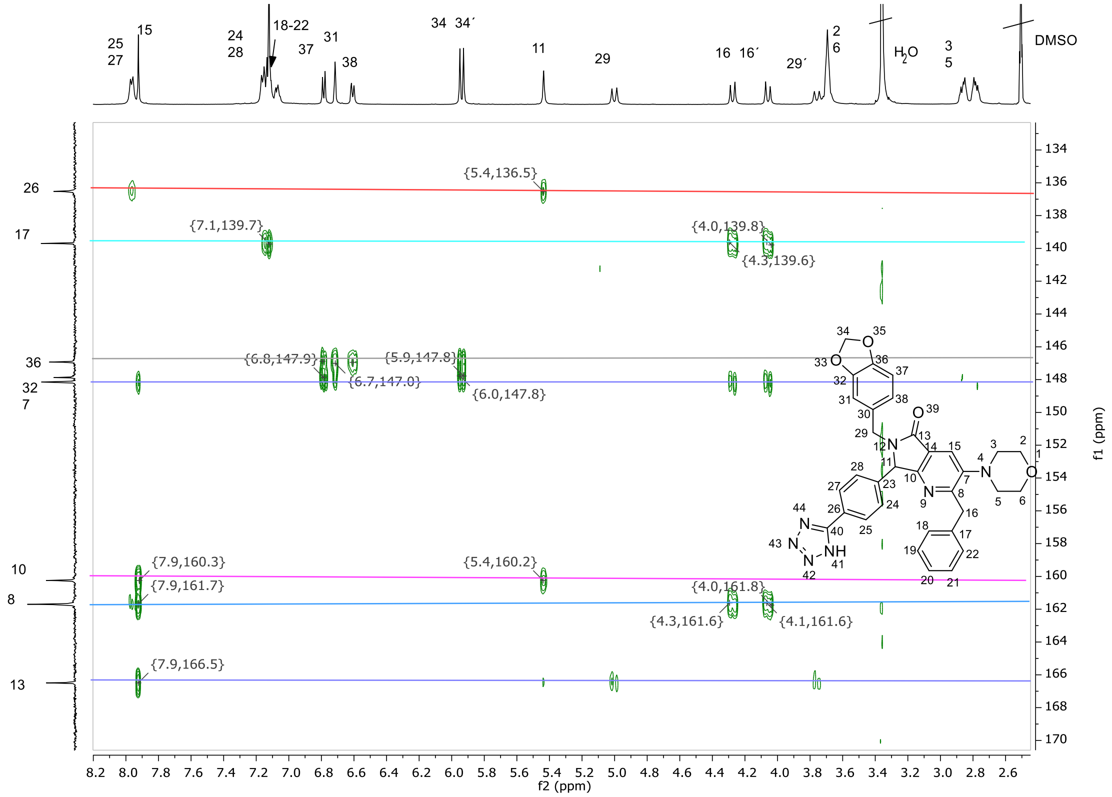

Figure S20. 2D-NMR (HMBC-part III) spectrum of the tetrazolyl-pyrrolo[3,4-*b*]pyridin-5-one **19**

Mass Spectrum SmartFormula Report

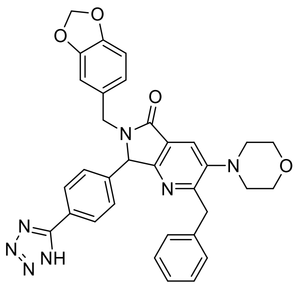

Chemical Formula: $C_{33}H_{29}N_7O_4$
Exact Mass: 587.23

Acquisition Parameter

Source Type	ESI	Ion Polarity	Positive	Set Nebulizer	0.3 Bar
Focus	Active			Set Dry Heater	180 °C
Scan Begin	50 m/z	Set Capillary	4500 V	Set Dry Gas	4.0 l/min
Scan End	3000 m/z	Set End Plate Offset	-500 V	Set Divert Valve	Waste

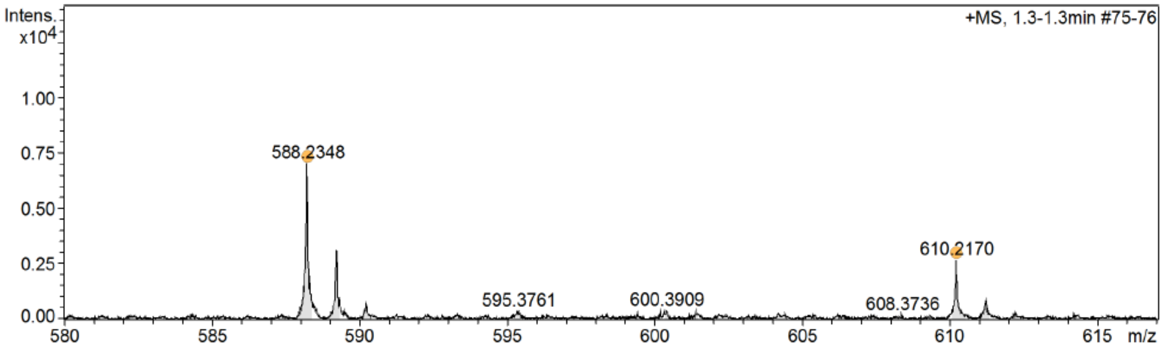

Meas. m/z	#	Ion Formula	Sum Formula	m/z	err [ppm]	mSigma	# Sigma
588.2348	1	C33H30N7O4	C33 H29 N7 O4	588.2354	-1.0	31.8	3
610.2170	1	C33H29N7NaO4		610.2173	-0.5	36.0	7

Figure S21. HRMS (ESI+-TOF) spectrum of the tetrazolyl-pyrrolo[3,4-*b*]pyridin-5-one **19**

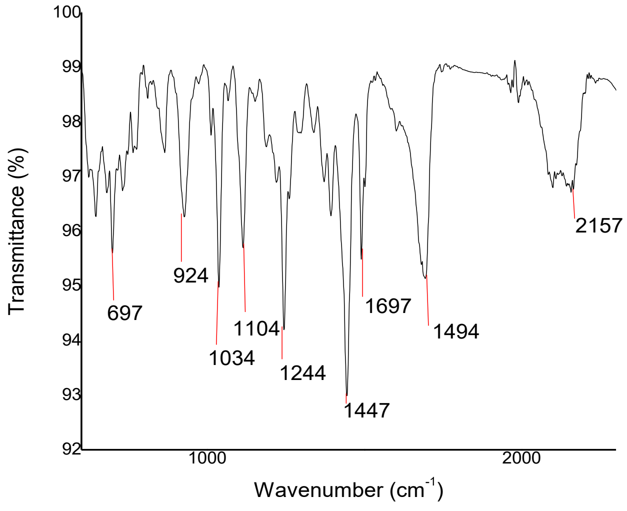

Figure S22. FT-IR (ATR) spectrum of the tetrazolyl-pyrrolo[3,4-*b*]pyridin-5-one **19**

Table S5. Synthesis of the 2,4-diamino-1,3,5-triazine-pyrrolo[3,4-*b*]pyridin-5-one **21** via a [4+2] cycloaddition.

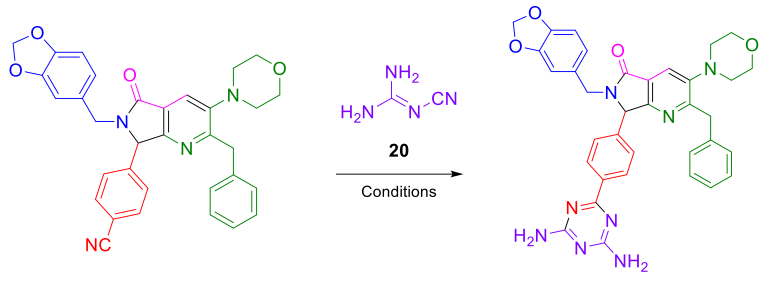

17 **21**

Entry[a]	Solvent	Temperature[b] (°C)	Time (h)	Yield[c] (%)
1	DMSO	100	2	--
2	DMSO	125	2	traces
3	DMSO	150	2	traces
4[d]	2-methoxyethan-1-ol	150	1.5	65

[a] KOH (8% mol) was the catalyst, and concentration in all inputs was [1.5 M]. [b] MW were the heating source.
[c] Calculated after purification by precipitation–filtration. [d] Optimal reaction conditions.

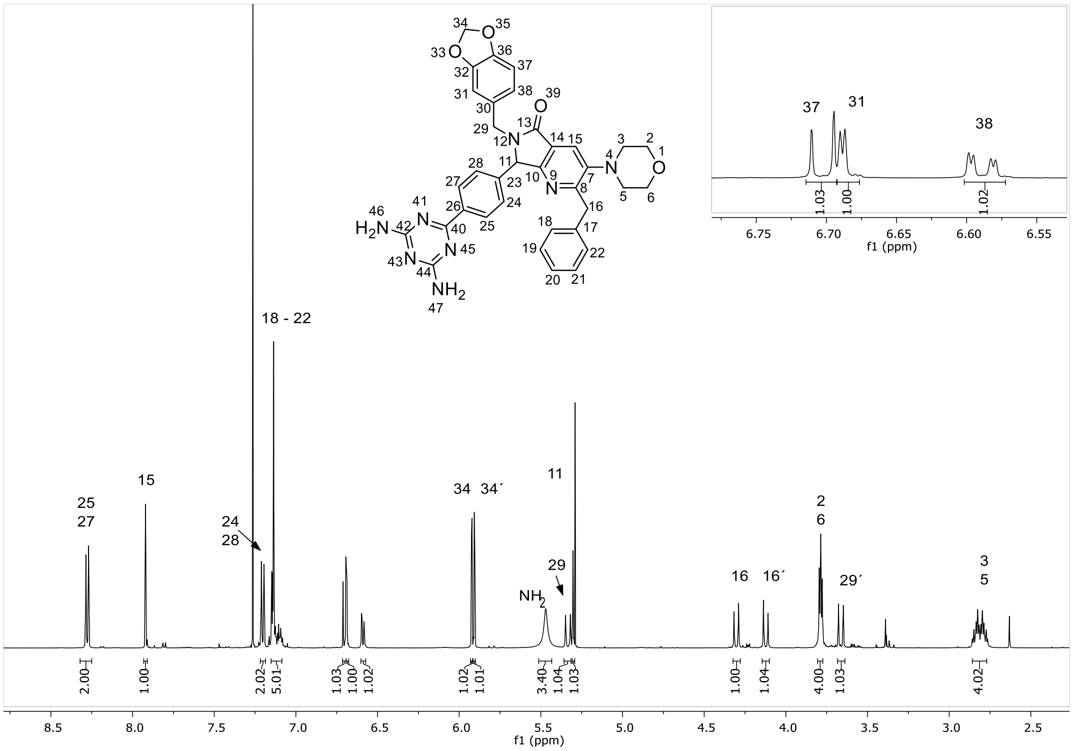

Figure S23. ^{1}H-NMR (500 MHz, $CDCl_3$) spectrum of the triazine-pyrrolo[3,4-*b*]pyridin-5-one **21**

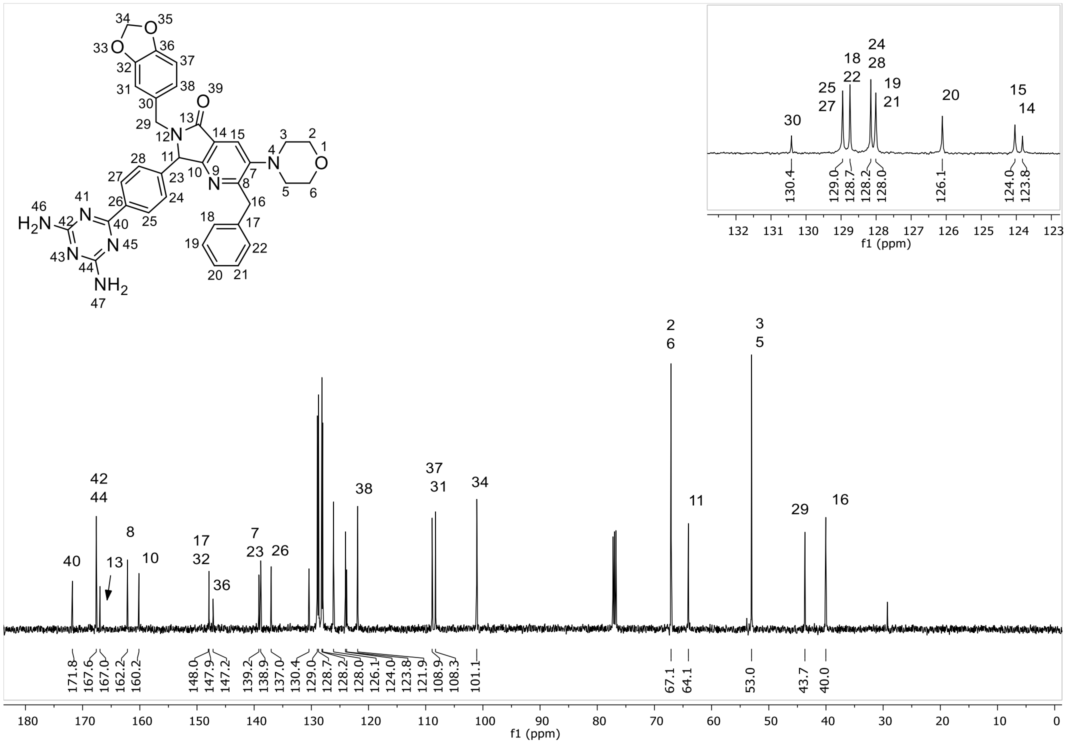

Figure S24. ^{13}C-NMR (125 MHz, $CDCl_3$) spectrum of the triazine-pyrrolo[3,4-*b*]pyridin-5-one **21**

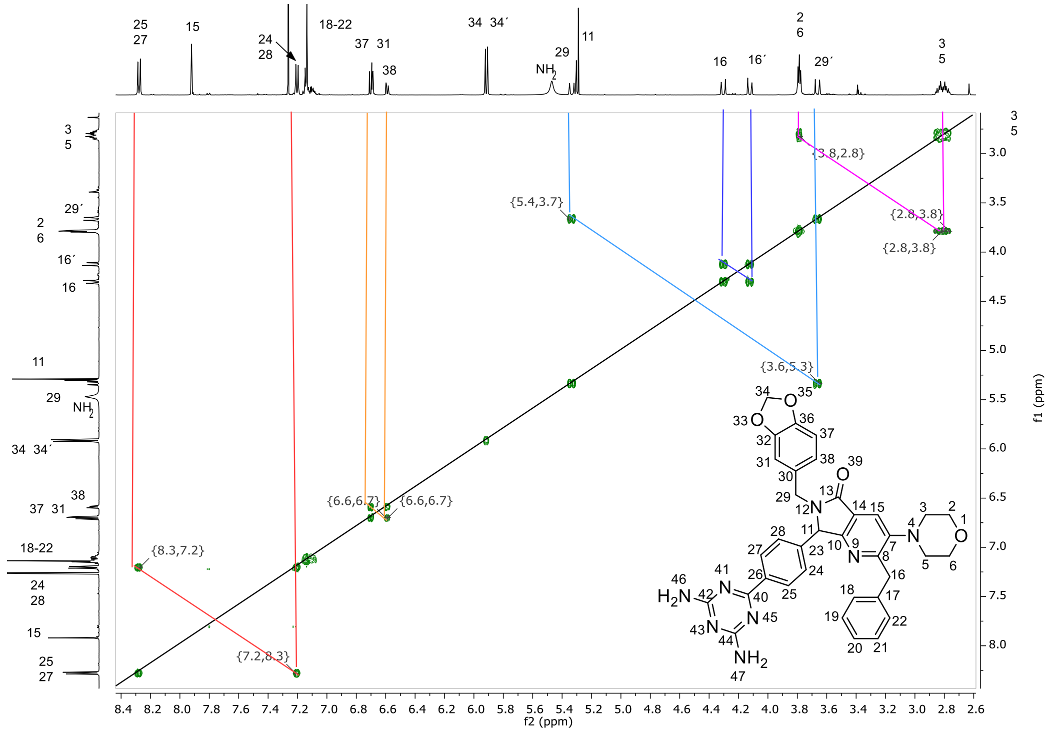

Figure S25. 2D-NMR (COSY) spectrum of the triazine-pyrrolo[3,4-*b*]pyridin-5-one **21**

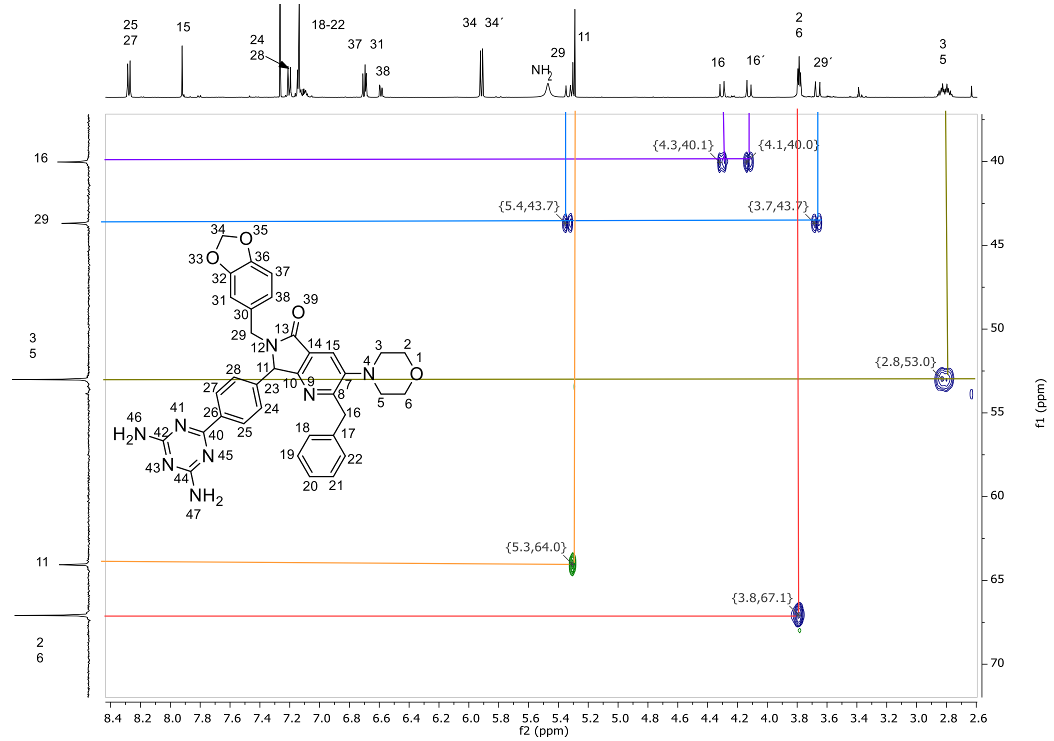

Figure S26. 2D-NMR (HSQC-part I) spectrum of the triazine-pyrrolo[3,4-*b*]pyridin-5-one **21**

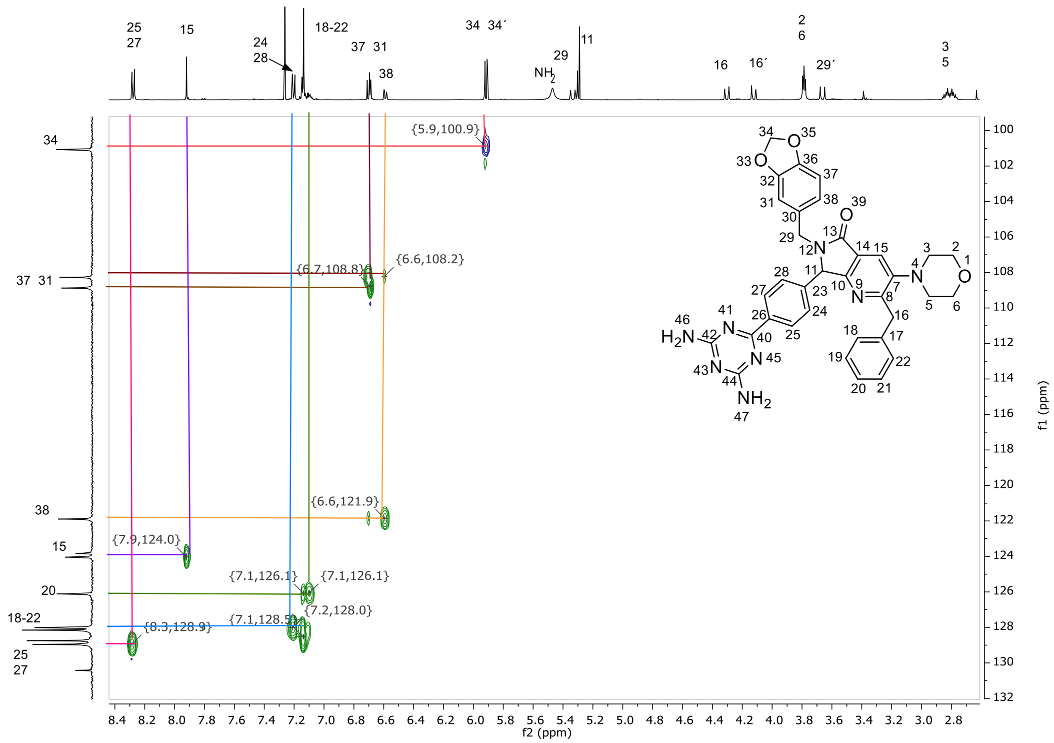

Figure S27. 2D-NMR (HSQC-part II) spectrum of the triazine-pyrrolo[3,4-*b*]pyridin-5-one **21**

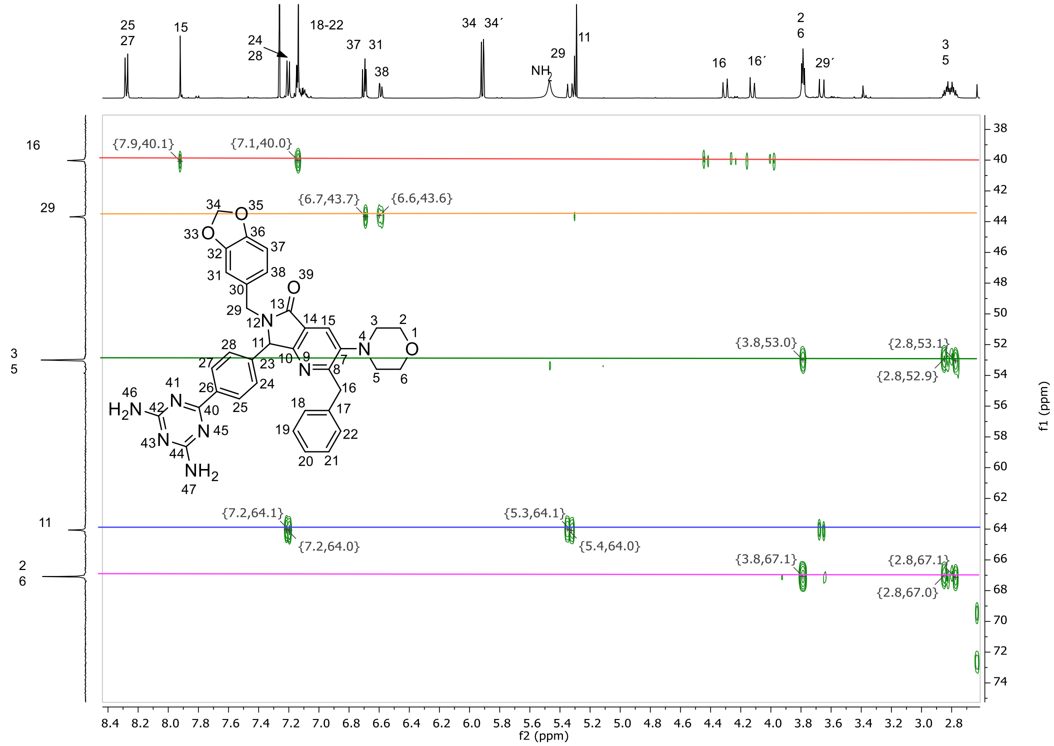

Figure S28. 2D-NMR (HMBC-part I) spectrum of the triazine-pyrrolo[3,4-*b*]pyridin-5-one **21**

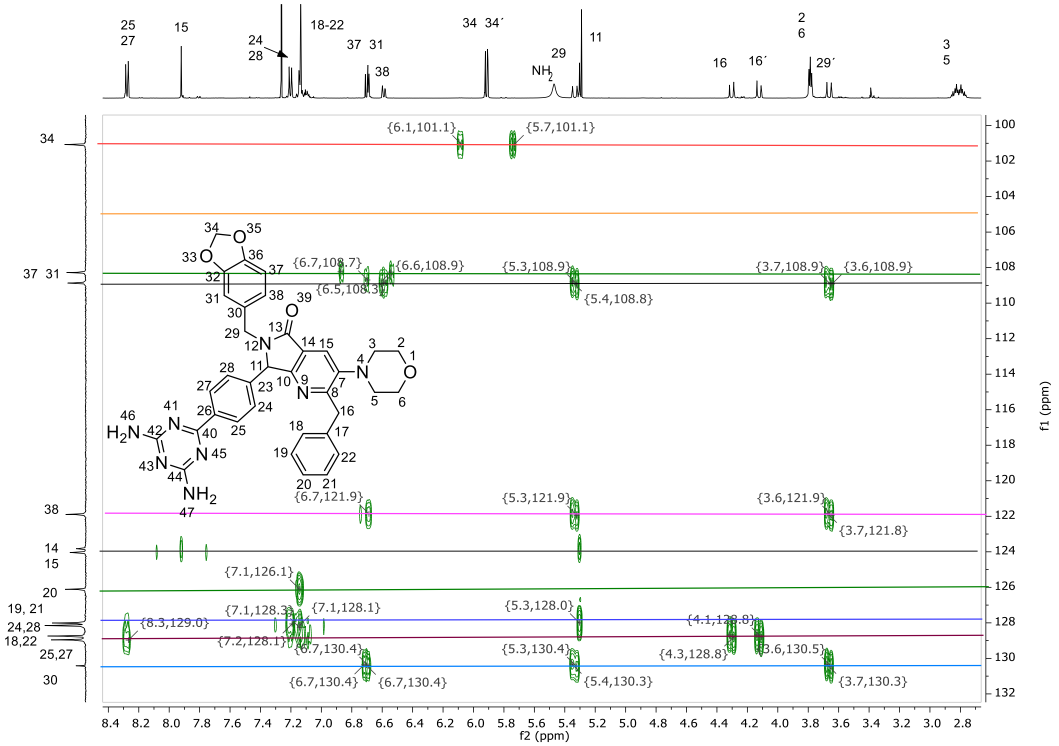

Figure S29. 2D-NMR (HMBC-part II) spectrum of the triazine-pyrrolo[3,4-*b*]pyridin-5-one **21**

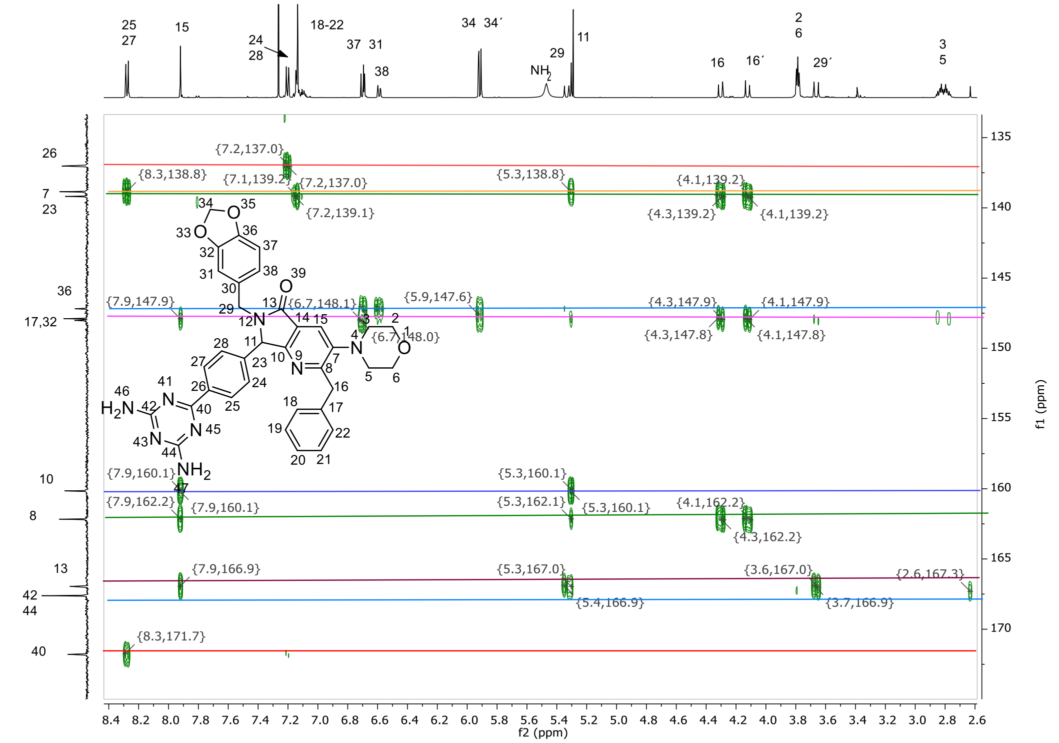

Figure S30. 2D-NMR (HMBC-part III) spectrum of the triazine-pyrrolo[3,4-*b*]pyridin-5-one **21**

Mass Spectrum SmartFormula Report

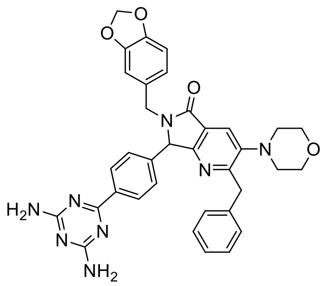

Chemical Formula: $C_{35}H_{32}N_8O_4$
Exact Mass: 628.25

Acquisition Parameter

Source Type	ESI	Ion Polarity	Positive	Set Nebulizer	0.3 Bar
Focus	Active			Set Dry Heater	180 °C
Scan Begin	50 m/z	Set Capillary	4500 V	Set Dry Gas	4.0 l/min
Scan End	3000 m/z	Set End Plate Offset	-500 V	Set Divert Valve	Waste

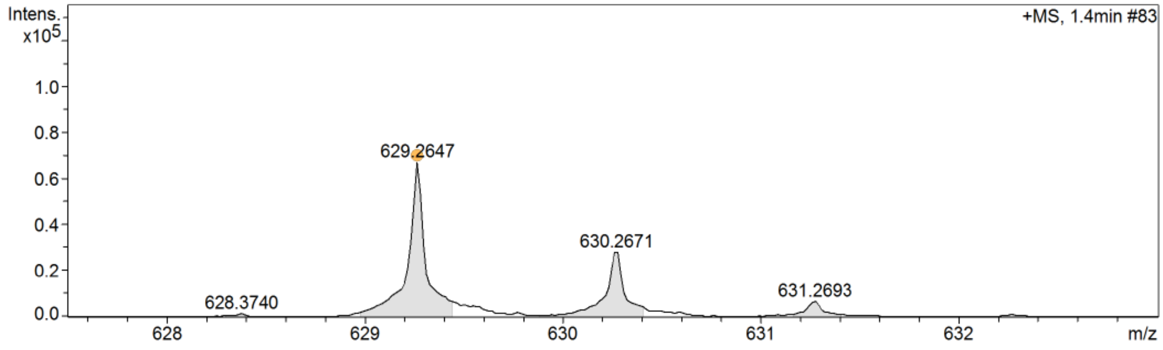

Meas. m/z	#	Ion Formula	Sum Formula	m/z	err [ppm]	err [mDa]	mSigma	# Sigma
629.2647	1	C35H33N8O4	C35 H33 N8 O4	629.2619	-4.4	-2.8	6.0	1
	1	C35H33N8O4	C35 H32 N8 O4	629.2619	-4.4	-2.8	6.0	1

Figure S31. HRMS (ESI+-TOF) spectrum of the triazine-pyrrolo[3,4-*b*]pyridin-5-one **21**

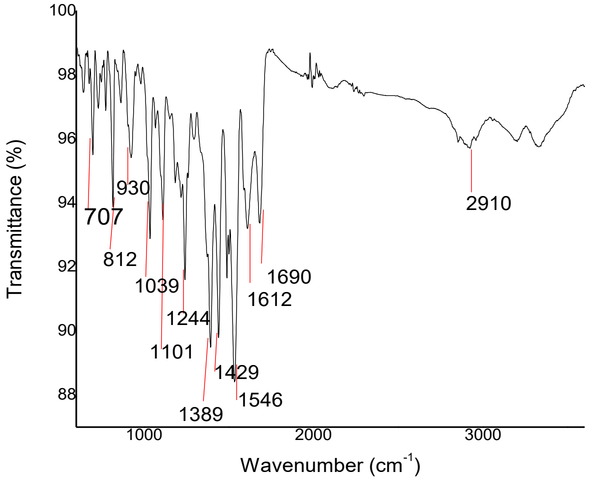

Figure S32. FT-IR (ATR) spectrum of the triazine-pyrrolo[3,4-*b*]pyridin-5-one **21**
